# Lignin-Derived Mesoporous Carbon for Sodium-Ion Batteries: Block Copolymer Soft Templating and Carbon Microstructure Analysis

**DOI:** 10.1021/acs.chemmater.3c01520

**Published:** 2023-12-06

**Authors:** Chantal Glatthaar, Mengnan Wang, Lysander Q. Wagner, Frederik Breckwoldt, Zhenyu Guo, Kaitian Zheng, Manfred Kriechbaum, Heinz Amenitsch, Maria-Magdalena Titirici, Bernd M. Smarsly

**Affiliations:** † Institute of Physical Chemistry, Justus-Liebig University, Heinrich-Buff-Ring 17, D-35392 Giessen, Germany; ‡ Department of Chemical Engineering, South Kensington Campus, Imperial College London, SW7 2AZ London, U.K.; § Center of Materials Research, Justus-Liebig University, Heinrich-Buff-Ring 16, D-35392 Giessen, Germany; ∥ Chemical Engineering Research Center, State Key Laboratory of Chemical Engineering, School of Chemical Engineering and Technology, Tianjin University, Tianjin 300072, China; ⊥ Institute of Inorganic Chemistry, Graz University of Technology, Stremayrgasse 9, A-8010 Graz, Austria; # Tohoku University Advanced Institute for Materials Research (AIMR), Chome-1-1 Katahira, Aoba Ward, Sendai, Miyagi 980-0812, Japan

## Abstract

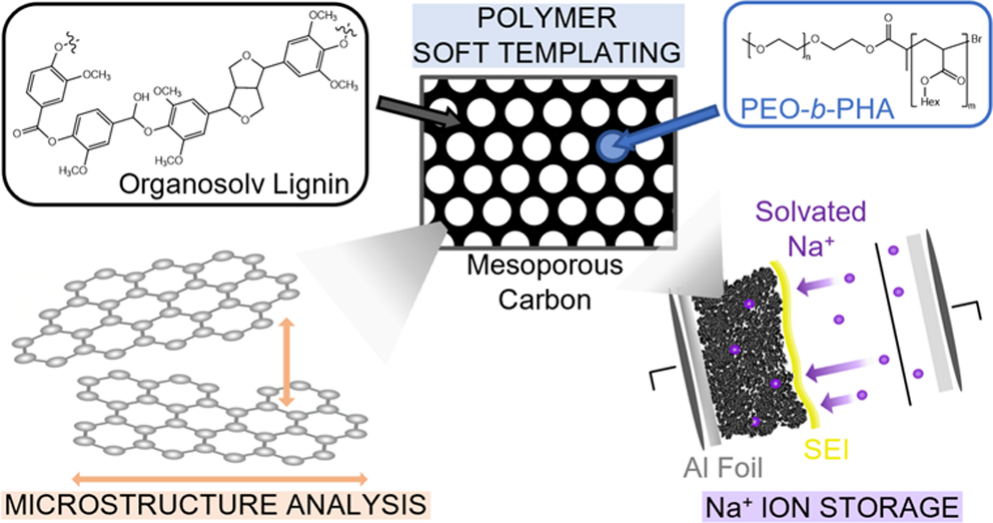

The demand for versatile and sustainable energy materials is on the rise, given the importance of developing novel clean technologies for transition to a net zero economy. Here, we present the synthesis, characterization, and application of lignin-derived ordered mesoporous carbons with various pore sizes (from 5 to approximately 50 nm) as anodes in sodium-ion batteries. We have varied the pore size using self-synthesized PEO_*n*_-*b*-PHA_*m*_ block copolymers with different PEO and PHA chain lengths, applying the “soft templating” approach to introduce isolated spherical pores of 20 to 50 nm in diameters. The pore structure was evaluated by transmission electron microscopy (TEM), nitrogen physisorption, and small-angle X-ray scattering (SAXS). We report the microstructure analysis of such mesoporous lignin-based carbons using Raman spectroscopy and wide-angle X-ray scattering (WAXS). In comparison with nontemplated carbon and carbons templated employing commercial Pluronic F-127 and PIB_50_-*b*-PEO_45_, which created accessible channels and spherical pores up to approximately 10 nm in diameter, the carbon microstructure analysis revealed that templating with all applied polymers significantly impedes graphitization upon thermal treatment. Furthermore, the gained knowledge of similar carbon microstructures regardless of the type of template allowed the investigation of the influence of different pore morphologies in carbon applied as an anode material in sodium-ion batteries, supporting the previous theories in the literature that closed pores are beneficial for sodium storage while providing insights into the importance of pore size.

## Introduction

Soft templating using amphiphilic polymers is a simple and suitable synthetic approach toward the production of mesoporous carbon materials, as initiated with the studies by Dai and co-workers.^1^ Properties such as a high specific surface area, good chemical and mechanical resistance, and a tunable pore structure enable a wide scope of different applications as in catalysis, adsorption, or energy storage, for instance.^2−4^ The evaporation-induced self-assembly (EISA) process is often applied to create an ordered mesophase of templates and precursors. Regarding this process, Clément Sanchez has brought significant scientific contributions to porous materials, especially by performing insightful mechanistic studies to reveal the underlying sol–gel chemistry of cross-linkable structures.^5,6^ Next to the feasible production of porous carbons, the importance of choosing sustainable bioderived and widely available precursors is key in the quest to produce sustainable energy materials. For example, Ghimbeu et al.^7^ substituted the carcinogen formaldehyde as a cross-linking agent in conventional synthetic strategies with phenol, resorcinol, or phloroglucinol as carbon precursors.^8^ Furthermore, Herou et al.^9^ used “organosolv lignin” as a bioprecursor to replace the fossil fuel derivate phloroglucinol in the synthesis of mesoporous carbon templated with Pluronic F-127. Applying lignin as a precursor exhibits obvious benefits, namely, high carbon content and large availability. Despite being the second most abundant natural polymer next to cellulose, lignin is rarely used in high-value applications such as the synthesis of advanced energy materials, for instance. It usually ends up as a waste product in the paper industry after the extraction of cellulose and is usually incinerated.^10,11^ Hence, the effective transformation of lignin to porous carbon materials, *e.g*., via templating approaches, is of growing interest. In most studies, soft templating approaches rely on templates that create relatively small mesopores rarely exceeding 10 nm in diameter. Apart from these, purposefully selected and synthesized block copolymers enable the generation of other pore geometries and sizes up to 40 nm.^12^ An increased mesopore diameter can be crucial for applications relying on enhanced mass transport, which is otherwise diffusion-limited within micropores or small mesopores.^13^ Although research on various block copolymers as soft templates has been carried out for several years, their utilization in large-scale applications is still hindered by often complex and time-consuming synthetic processes. Recent studies by Wagner et al.,^13^ as well as the initial work by Lokupitiya et al.,^14^ revealed that PEO_*n*_-*b*-PHA_*m*_ (poly(ethylene oxide)_*n*_-*b*-poly(*n*-hexyl acrylate)_*m*_ with *n* and *m* as numbers of the respective repeating units) block copolymers can be suitable templates for various metal oxides creating large mesopores, which is a challenge to be introduced by soft templating without additional pore size swelling agents. Their synthesis via a supplemental activator reducing agent atom transfer radical polymerization (SARA ATRP) enables facile adjustment of different block chain lengths and is feasible with standard laboratory equipment, which represents a major advantage. In this study, the applicability of PEO_*n*_-*b*-PHA_*m*_ block copolymers as templates for lignin-derived carbons with different pore diameters due to varying block lengths is evaluated. As previous investigations are restricted to PEO_*n*_-*b*-PHA_*m*_ templating of metal oxides, here we extend the versatile application of PEO_*n*_-*b*-PHA_*m*_ block copolymers also for mesoporous carbon next to the templating of metal oxides.

Since reduced solubility comes with an increased molecular weight of polymers, which is necessary to produce large pore diameters, precipitation or phase separation would obstruct the EISA mechanism. Moreover, the thermodynamic and kinetic stability of block copolymer micelles formed within the EISA process is necessary to resist polymerization reactions during heat-induced precursor cross-linking. Hence, a rational choice of the solvent and reaction conditions becomes a crucial challenge when large block copolymers are used as templates in contrast to the straightforward application of commercial templates from the Pluronic family. Additionally, as lignin exhibits a highly branched molecular structure and high polydispersity, a uniform arrangement around large self-arranged polymer micelles during the EISA process is key.^15^ To explore the general possibility of introducing large mesopores in carbons based on a precursor mixture of phloroglucinol and organosolv lignin in a mass ratio of 1:1, PEO_*n*_-*b*-PHA_*m*_ block copolymers were employed in this study. Further, the comparison with two additional types of block copolymers (Pluronic F-127 and PIB_50_-*b*-PEO_45_) as templates, all applied in a mass ratio of 1:1 between the template and carbon precursors, aims to investigate the difference in accessibility, shape, and size of generated pores due to different soft templates for application as an anode material in sodium-ion batteries.

In addition to investigating the pore morphology based on the type of block copolymer, the effect of the block copolymer template on carbonization and graphitization behavior is also evaluated. The nongraphitizable character and microstructure of conventional nontemplated resin-based carbons have been well investigated already.^16−18^ The absence of crystalline long-range order within the graphene layer stacking and structural disorder of the two-dimensional graphene lattice cause broad and asymmetric (*hk*) and (00*l*) reflections in the wide-angle X-ray scattering (WAXS) pattern instead of general (*hkl*) reflections. Hence, conventional line-width-based microstructure evaluation of nongraphitic carbons is hindered and not meaningful.^18,19^ Therefore, applying the algorithm by Ruland and Smarsly^20^ has proved itself as a useful tool for nongraphitic carbon microstructure analysis in previous studies.^16,18,19,21^ However, due to the novelty of the synthetic approach of producing carbons from organosolv lignin, the development and structural features of the microstructure of lignin-derived carbons have not been investigated yet. Inspired by Clément Sanchez and his pursuit of a detailed analysis and materials characterization, this study aims to present a starting point for enlightening the microstructure of templated lignin-derived hard carbons. This provides an initial understanding of the carbonization behavior of lignin isolated in the organosolv process. Also, using varying block copolymers as templates compared to nontemplated lignin-based carbon enables us to investigate the impact of different templates on carbonization behavior. This offers a door opener for follow-up studies of carbon analysis of different synthesis and precursor systems.

The resulting carbon materials from this study with different pore sizes, pore accessibility, and graphitization levels were applied as anode materials in sodium-ion batteries (SIBs), the next generation of commercial and more sustainable batteries to complement lithium-ion batteries. Hard carbons, as, *e.g*., our synthesized mesoporous lignin-based carbon materials are the most utilized types of carbons for anodes in SIBs. However, their sodium storage mechanism is still under debate. The latest theories reported in the literature support a combination of adsorption–desorption of Na^+^ at defects and intercalation in the sloping region of the charge/discharge curve and a pore-filling mechanism in the plateau region, which needs closed pores.^22,23^ Based on these theories, to maximize sodium storage, an interlayer spacing above 3.5 to 3.6 Å and closed pores are necessary. Since the determination of the exact value of this important structural parameter from WAXS data is impeded by the translational disorder in the stacking, the fitting approach by Ruland and Smarsly^20^ was applied to obtain as reliable values as possible, which we compared to WAXS standard analysis (Scherrer-type analysis). The larger the pore dimension, the higher the plateau capacity should be, provided that the interlayer spacing stays within the mentioned range. For carbons with open porosity, the Coulombic efficiency should be low due to extensive electrolyte decomposition on the high surface area provided by open pores. As for formality, it should be noted that the terminology of “closed pores” will be continued instead of “voids”, as accessibility for sodium ions is still given here. Applying PEO_*n*_-*b*-PHA_*m*_ polymers as templates for these lignin-derived hard carbons with both open and closed pores and a similar carbonization degree leading to a rather similar graphitic microstructure validates this previous sodium storage mechanism. These are just preliminary results since the focus here was the synthesis and characterization of this new type of porous lignin-derived carbons, but the ability to control closed vs open pores, their pore size, and pore volume opens new avenues for a better validation of the sodium-ion storage mechanism in hard carbons.

## Experimental Methods

### Materials

Phloroglucinol (1,3,5-benzentriol, ≥99.0%), the triblock copolymer Pluronic F-127 (poly(ethylene oxide)_*x*_-poly(propylene oxide)_*y*_-poly(ethylene oxide)_*x*_, *M*_W_ = 12.6 kDa), and methanol (≥99.8%) were obtained from *Sigma-Aldrich*. Aqueous glyoxal solution (40 wt %) was purchased from *Alfa Aesar*. Chemicals were used as received without any further treatment. Organosolv lignin extracted from beech wood was provided by the Fraunhofer Center for Chemical-Biotechnological Processes (CBP) in Jena. Further information on lignin characterization and extraction can be found elsewhere.^24^ The diblock copolymer PIB_50_-*b*-PEO_45_ (poly(isobutylene)-*block*-poly(ethylene oxide)) was purchased from *BASF* (*Ludwigshafen, Germany*), while the diblock copolymers PEO_*n*_-*b*-PHA_*m*_ (poly(ethylene oxide)-*b*-poly(*n*-hexyl acrylate)) were synthesized as described below.

### Synthesis of PEO_*n*_-*b*-PHA_*m*_ Diblock Copolymers

The two-step synthesis was originally reported by Lokupitiya et al.^14^ According to Wagner et al.,^13^ PEO-Br as a halide-capped macroinitiator was synthesized by esterification of α-methyl-ω-hydroxy poly(ethylene oxide). In a subsequent supplemental activator reducing agent atom transfer radical polymerization (SARA ATRP), PEO_*n*_-*b*-PHA_*m*_ copolymers of various block lengths were obtained. Details on PEO_*n*_-*b*-PHA_*m*_ diblock copolymer synthesis can be found in the recent literature.^13^Supporting Information (SI) provides an overview of the used amounts of starting materials to synthesize respective polymers (Table S1) and polymer characterization by ^1^H NMR (Figures S1–S6), gel permeation chromatography (GPC) (Figure S7), and dynamic light scattering (DLS) (Figure S8).

### Preparation of Carbon Materials

The procedure in the initial report of lignin-derived mesoporous carbons by Herou et al.^9^ has been adjusted as follows: The synthesis of the mesoporous carbon materials was carried out by applying an EISA process. First, the respective block copolymer (2.25 g) was dissolved in methanol by ultrasonication (45 kHz, 70 °C). For Pluronic F-127 and PIB_50_-*b*-PEO_45_ as templates, polymer dispersions were obtained in 62.5 mL of methanol in a glass beaker. Higher molecular mass causing poorer solubility required doubling the volume of solvent (125 mL of methanol) to dissolve the PEO_*n*_-*b*-PHA_*m*_ diblock copolymers. One after another, the carbon precursors, lignin (0.65 g) and phloroglucinol (0.65 g), and aqueous glyoxal solution (1.25 mL) as a cross-linker were added after the complete dissolution of each by ultrasonication. In the case of PEO_214_-*b*-PHA_322_ polymer dispersion, an additional 30 mL of methanol was necessary to dissolve lignin completely. Covering the beakers prevented undesired solvent evaporation during ultrasonic treatment. Afterward, the solutions were left open in order to evaporate methanol at 40 °C for one night. Shiny, hard, and brown solids were obtained in each beaker. The following thermopolymerization was initiated upon thermal treatment at 85 °C overnight. The solids obtained were transferred to crucibles. Template removal at 350 °C and carbonization at 900 °C under an inert atmosphere (*STF 16/180* from *Carbolite Ltd.*; N_2_ flow rate of 500 mL min^–1^; heating rate of 1 °C min^–1^; 1 h dwelling time at 350 °C; further temperature increase with 1 °C min^–1^; 2 h dwelling time at 900 °C, natural cooling) yielded the respective carbon material as the product. Reactant mass ratios of carbon precursor: template: aqueous glyoxal and phloroglucinol: lignin were chosen according to the previous literature^9^ and set to 1:1.731:1.216 and 1:1, respectively. To investigate the influence of carbonization temperature on carbon microstructure and porosity, carbonization at 1300 °C (*STF 16/180* from *Carbolite Ltd.*; N_2_ flow rate of 500 mL min^–1^; heating rate of 5 °C min^–1^; 2 h dwelling time; natural cooling) was carried out as post-treatment.

### Preparation of Carbon Anodes

The carbon materials were ground using a mortar and pestle to break down the monolithic structure and homogenize the particle size. After that, the carbon powder was mixed with the binder carboxymethyl cellulose (*Sigma-Aldrich*) in a mass ratio of 9:1 using a mortar and pestle for 30 min to prepare an aqueous slurry. The hard carbon electrodes were then coated by using the mixed slurry onto Al foil (*MTI Corporation*). Before being cut into pieces for the further assembly of coin cells, the coated Al foil was dried at room temperature, followed by 80 °C in a vacuum oven overnight. The loading mass of the coated electrodes (0.8 cm × 0.8 cm) was controlled within a range of 1.5–2.0 mg cm^–2^.

### Electrochemical Measurements

The electrochemical testing was conducted using stainless steel coin cells (CR2032, *MTI Corporation*) at room temperature, and the cell assembly was completed in an Ar-filled glovebox (H_2_O, O_2_ < 0.5 ppm, *mBraun*). The commercial electrolytes of 1 M NaPF_6_ in mixed solvents, ethylene carbonate–diethyl carbonate (EC–DMC, 1:1 vol %, *Elyte*), were used. In the half-cell configuration, a piece of sodium metal (12 mm in diameter, *Alfa Aesar*) was used as both the counter and the reference electrodes. The electrodes were placed against the sodium metal with a glass fiber separator (16 mm in diameter, GF/A Glass microfiber filters, *Whatman*) soaked with 100 μL of electrolytes. The half cells were discharged and charged between 0.001 and 2.5 V vs Na^+^/Na at different current densities of 0.1, 0.2, 0.5, 1.0, and 2.0 C (1 C = 300 mA g^–1^) as well as cycling at 0.5 C.

### Galvanostatic Intermittent Titration Technique (GITT)

Before the GITT measurement, the samples were cycled at a constant current of 0.1 C for the first cycle. This precycle was to exclude the influence of SEI formation. From the second cycle, a sample was galvanostatically discharged for 10 min and then rested for 80 min until the potential reached 1 mV (V vs Na^+^/Na). The discharge current was based on the reversible capacity to ensure similar numbers of data points across different samples. For example, the reversible capacities of the PIB_50_-*b*-PEO_45_-templated carbon and the PEO_428_-*b*-PHA_265_-templated carbon are around 100 and 220 mAh g^–1^, respectively. Then, the discharge currents were 10 and 22 mAh g^–1^. According to the potential gap between equilibrium potential and nonequilibrium potential under applied current, the diffusivity coefficient can be estimated using Fick’s second law with the following simplified equation^25^
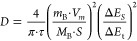
where τ is pulse time length, *m*_B_, *M*_B_, and *V*_M_ are the active mass, the molar mass, and the molar volume of carbon, respectively, and *S* is the active area of the working electrode (in this work, the area of the electrode was used in the calculation), Δ*E*_S_ and Δ*E_t_* can be obtained from a GITT curve (see Figure S9 in the SI for a representative curve in GITT).

### Characterization of PEO_*n*_-*b*-PHA_*m*_ Polymers

The hydrodynamic diameter was determined by DLS experiments carried out on a *Litesizer 500* from *Anton Paar*. Polymer dispersions used in the synthesis procedure were diluted 4-fold in a disposable measurement cell. Measurement was performed in three runs of ten seconds each run and evaluated with the software *Anton Paar Kalliope Professional 2.18.1*. Proton-nuclear magnetic resonance (^1^H NMR) experiments were performed at 25 °C on a *Bruker Avance II 400 MHz*, *Bruker Avance III 400 MHz HD*, and *Bruker Ascend Advance IV Neo 7*. The spectra were referenced to the solvent peak at 7.27 ppm and evaluated using *MestReNova 14.1.2*. Tetrahydrofuran with a flow rate of 0.5 mL min^–1^ served as an eluent for GPC. Detection with simultaneous UV (*TSP UV 2000*) and refractive index (*Shodex RI-101*) took place at 25 °C. Calibration was done with poly(styrene) standards (*PSS, Mainz, Germany*). A 300 mm × 8 mm *PSS SDV linear M* column was applied as a stationary phase and packed with 3 μm particles with 10^2^ to 10^6^ Da of mass range. An injection volume of 100 μL was filtered through 0.45 μm filters beforehand and contained about 0.15 wt % polymeric sample.

### Characterization of Carbon Materials

Nitrogen physisorption experiments were performed with *TriStar II PLUS* from *Micromeritics* at 77 K. Data evaluation was carried out with the affiliated software *TriStar II PLUS 3.00*. For pore size distribution determination, a nonlocal density functional theory (NLDFT) kernel for carbon with cylindrical pore geometry was applied to the adsorption branch. Degassing of the carbon samples was accomplished at 220 °C for 16 h with *Micromeritics′s Smart VacPrep* and the *Smart VacPrep* software. Transmission electron microscopy (TEM) was carried out for the direct imaging of carbon materials in a *JEOL JEM-2100F* at 200 kV, and the images were analyzed with *DigitalMicrograph* 3.52.4932.0 from *Gatan*. The carbon powders were dispersed in acetone by sonicating for 15 min and left to settle for 10 min. The upper solution was collected and deposited onto the TEM grids (Holey Carbon Films on 300 Mesh Copper Grids, *Agar Scientific*). The grid was then left to dry overnight and stored in dry conditions before imaging. A Scanning Electron Microscope (*LEO Gemini 1252 FEG-SEM*) was used to study the morphologies of samples operating at an accelerating voltage of 3 keV and a working distance of around 3 mm. Before the measurement, the samples were secured onto the alumina sample holders by using conductive double-sided carbon tapes. Wide-angle X-ray scattering (WAXS) measurements were performed with an *X′Pert Pro MPD* diffractometer from *PANalytical* in Bragg–Brentano geometry at room temperature. Cu-*K*_α1_ radiation (λ = 1.5406 Å) and Cu-*K*_α2_ radiation (λ = 1.5444 Å) in a 1:1 ratio at 40 kV and 40 mA were applied. Measurements were operated with a step size of 0.08° in the range of 5° < 2θ < 115°. Data analysis was based on the algorithm of Ruland and Smarsly^20^ implemented in the *OctCarb* script by Osswald and Smarsly^21^ and executed by the GNU *Octave* software. Details about the mathematical evaluation of quantitative microstructure parameters from the whole WAXS pattern of nongraphitic carbons can be found elsewhere.^18−20^ Small-angle X-ray scattering (SAXS) was operated with a *SAXSpoint 2.0* instrument from *Anton Paar* equipped with a *Dectris EIGER2 R 1M* hybrid pixel area X-ray detector and a microsource creating point-focused and slit-collimated Cu–K_α1_ (λ = 1.54 Å) radiation at 50 W. The sample holder from *Anton Paar*, on which the sample plate was fixed with a vacuum-tight sealing tape at both sides, at a motorized X/Y table consisted of a 0.4 nm thick metal plate with 20 square holes (4 mm × 4 mm with 10 mm center-to-center distances). Sample-to-detector distance was set to 575.65 mm. Measurement was carried out with 25 runs for each sample in a vacuum (1 mbar) and at 25 °C temperature. Each run contained an exposure time of 2 min. On the one hand, SAXS data evaluation was carried out with chord length distribution (CLD) analysis according to Stoeckel et al.^26^ On the other hand, the software *SASfit* 0.94.12 was applied to fit the SAXS patterns, in which the implemented decoupling approximation described the fitting model with a lattice parameter and a form factor for spheres with a Gaussian size distribution. In addition, modeling SAXS patterns assuming a face-centered cubic (FCC) array of polydisperse spheres was accomplished with the software *Gnuplot*. Applying a laser excitation wavelength of λ = 532 nm and a power of 6.25 mW, Raman spectra were recorded with a *Senterra II* instrument by *Bruker*. Data export was carried out with *Opus 8.1*. Density measurements were carried out with a *Micromeritics AccuPyc II 1340 Gas Pycnometer*, which was operated with the software *AccuPyc II 1340 V2.00*. A 1 mL volume cuvette for the analyzed sample, 25 °C setting temperature, and helium as working gas were used. The procedure consisted of 20 purges over 50 cycles. The results for the last 30 cycles were applied to obtain an average density value.

## Results and Discussion

**Figure 1:**
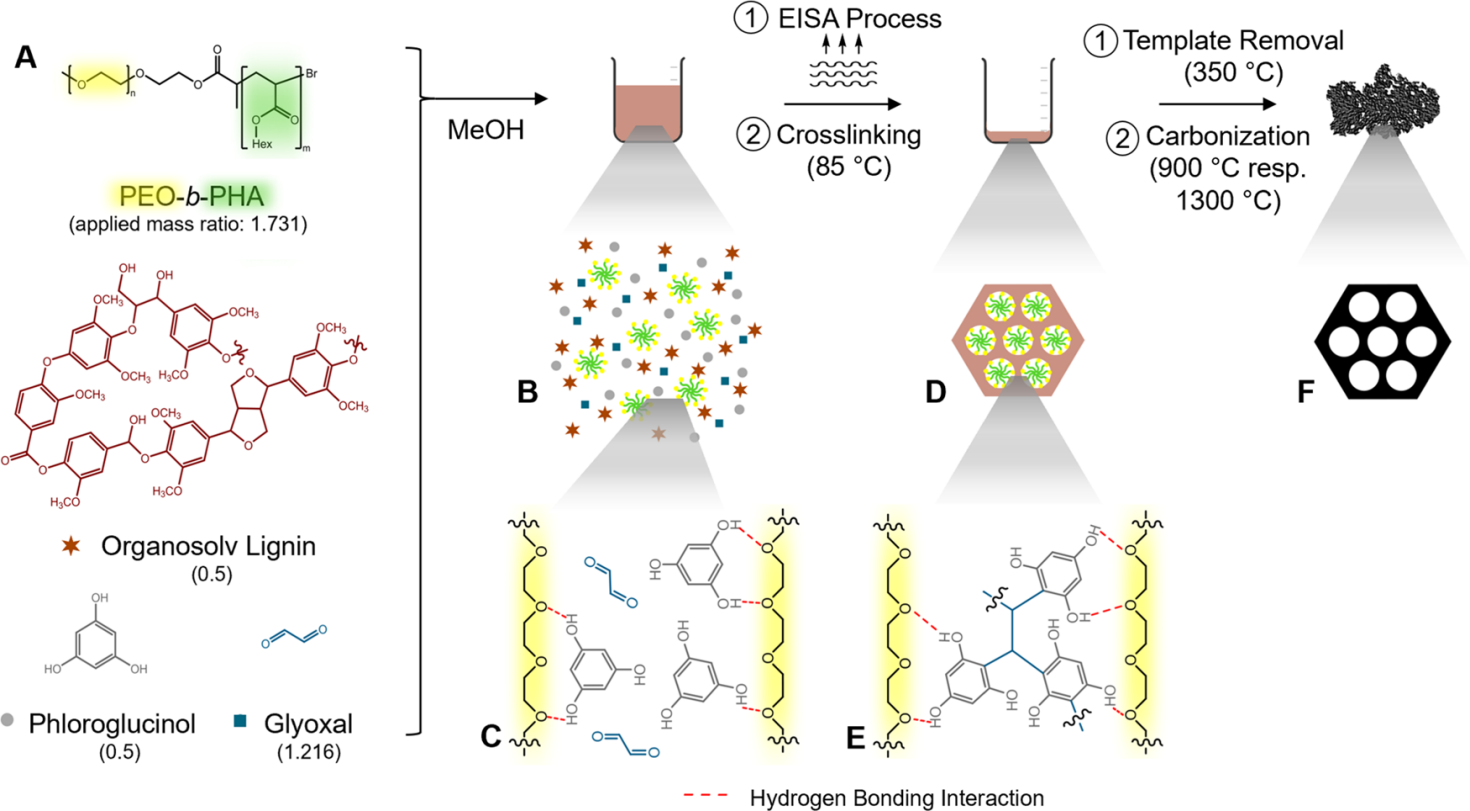
Schematic synthetic procedure at macroscopic and molecular level. (A) Molecular structure of PEO_*n*_-*b*-PHA_*m*_ block copolymers. (B) Schematic representation of initial precursor dispersion with fixed mass ratios of template, carbon precursors, and aqueous glyoxal solution (1.731:1:1.216) and (C) molecular assembly due to hydrogen bonding interaction. Furthermore, (D) ordered mesophase after the EISA process and thermopolymerization with (E) the resulting covalent bonding. (F) Mesoporous carbon after carbonization at 900 °C, respectively 1300 °C, in a nitrogen atmosphere.

The synthesis of mesoporous carbons was carried out by soft templating with fixed mass ratios of template, carbon precursors, and aqueous glyoxal solution (1.731:1:1.216) as well as within the carbon precursors phloroglucinol and organosolv lignin (1:1).^9^ While all other synthetic conditions were maintained identically, different block copolymers were applied as templates to test their applicability for soft templating of lignin-based carbons and to achieve mesoporosity with controlled pore size and shape. Next to the well-established triblock copolymer Pluronic F-127,^9,27,28^ the diblock copolymer PIB_50_-*b*-PEO_45_ (poly(isobutylene)-*block*-poly(ethylene)) and three PEO_*n*_-*b*-PHA_*m*_-type (poly(ethylene oxide)-*b*-poly(*n*-hexyl acrylate)) polymers (Figure 1A) with varying block lengths (both *n* and *m*) are applied as templates within the synthetic procedure, which is schematically illustrated in Figure 1, yielding mesoporous carbons.

After the dissolution of the polymer template in methanol, organosolv lignin, phloroglucinol, and glyoxal are subsequently added to the polymer dispersion (Figure 1B). Due to hydrogen bonding, carbon precursors possessing a hydroxy moiety interact with the PEO sequence of the templates, as indicated in Figure 1C, taking the example of phloroglucinol as a precursor. These noncovalent interactions govern the subsequent organic–organic self-assembly process.^8^ Driven by the mutual incompatibility of hydrophilic and hydrophobic polymer blocks, the polymers form micelles with the hydrophobic sequence (e.g., PPO, PIB, or PHA), building the core of a micelle in methanol as a hydrophilic solvent. Note that PIB-*b*-PEO-type and PEO-*b*-PHA-type block copolymers are present as micelles already at minute concentrations, while in the case of Pluronic F-127, micelles only form at elevated concentrations upon solvent evaporation. For all three types of block copolymers, microphase separation is promoted within the EISA process, and thermopolymerization of precursor monomers at 85 °C stabilizes the ordered mesophase (Figure 1D) by cross-linking reactions with glyoxal, thereby generating covalent carbon-bridged phenols (Figure 1E).^27,29^ The three-dimensionally interconnected network enables subsequent template removal without collapsing mesostructures, and carbonization at 900 °C, respectively 1300 °C, yields the final mesoporous carbon (Figure 1F).^29^

In order to relate the finally obtained mesopore dimensions to the block copolymer micelles, solutions of the block copolymers in methanol were studied in dynamic light scattering (DLS) measurements. As shown in Figure S8, for PIB_50_-*b*-PEO_45_ and PEO_*n*_-*b*-PHA_*m*_ polymers, colloidal objects with well-defined and quite large hydrodynamic radii are observed (see Table 1), which can be attributed to micelles, in accordance with the DLS studies of Sarkar et al.^30^ It is important to note that for these two types of block copolymers, the minimized exchange dynamics of persistent micelle templates with preserved micelle dimensions allow control of yielded templated materials and defined mesoporosity.^30,31^ In addition, the diameters increase with extended block lengths, which is in accordance with the general principles of micelle formation. As a result, the chosen combination of methanol as a solvent and PEO_*n*_-*b*-PHA_*m*_ block copolymers enables control of defined mesoporosity by micelle templating.

**Table 1:** Comparison of Hydrodynamic Micelle Diameters and Pore Size in Resulting Mesoporous Carbon

template	pluronic F-127	PIB_50_-*b*-PEO_45_	PEO_251_-*b*-PHA_95_	PEO_428_-*b*-PHA_265_	PEO_214_-*b*-PHA_322_
hydrodynamic micelle diameter/nm	3 ± 1	15 ± 3	31 ± 5	69 ± 10	91 ± 15
mean carbon mesopore diameter (TEM)/nm^a^	5 ± 1	9 ± 1	23 ± 4	37 ± 6	52 ± 22
mean carbon pore wall thickness (TEM)/nm^a^		4 ± 1	5 ± 2	7 ± 2	9 ± 3
range of mean mesopore diameters (nitrogen physisorption)/nm^b^	4–7	8–15	n.a.	n.a.	n.a.
mean mesopore diameter (SAXS)/nm^c^			22 ± 3	37 ± 5	

a Obtained by averaging TEM measurements.

b Pore diameter evaluation of carbons templated with PEO_*n*_-*b*-PHA_*m*_ polymers is not applicable, as explained later (see Figure 3B).

c Obtained by fitting procedure applying a Percus–Yevick decoupling approach.^32^

**Figure 2:**
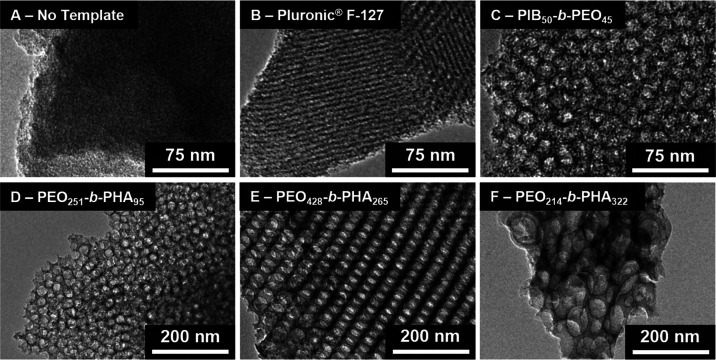
TEM images display yielded carbons after carbonization at 900 °C synthesized (A) without any template, (B) with Pluronic F-127, and (C) with PIB_50_-*b*-PEO_45_ as a template. In addition, PEO_*n*_-*b*-PHA_*m*_ polymers with varying block lengths (D) *n* = 251 and *m* = 95, (E) *n* = 428 and *m* = 265, and (F) *n* = 214 and *m* = 322 served as templates.

Accordingly, TEM micrographs (Figure 2) reveal the successful generation of mesoporous lignin-derived carbon by soft templating using three types of block copolymers. Lignin-based carbon templated with Pluronic F-127 exhibited characteristic channel pores (Figure 2B) as previously produced by Herou et al.^9^

PIB_50_-*b*-PEO_45_ (Figure 2C) and PEO_*n*_-*b*-PHA_*m*_ (Figure 2D–F) induced arrays of spherical pores with average diameters of 9 nm (PIB_50_-*b*-PEO_45_) and 23 to 52 nm (PEO_*n*_-*b*-PHA_*m*_). For the latter, the pore diameters increased with growing polymer block lengths and are thus in relation to the hydrodynamic micellar diameters (Table 1**)**. Hence, for PEO_*n*_-*b*-PHA_*m*_ polymers, the PHA block determined predominantly the resulting pore diameter, comparing PEO_251_-*b*-PHA_95_ and PEO_214_-*b*-PHA_322_, featuring similar PEO blocks.^13^ As the determination of the hydrodynamic diameter encompasses the micelle core, the micelle corona, and a solvation sphere, it is reasonable to expect systematically smaller resulting pore sizes. These sizes predominantly depend on the micelle core. This expectation is particularly applicable when considering PIB50-b-PEO45 and PEOn-b-PHAm polymers as templates.^30^ The diameter of mesopores for Pluronic F-127 templated carbon is in line with the comparably small hydrodynamic radius.

Carbons synthesized without any template do not reveal any mesoscale porosity, thus proving that the mesoporosity is a consequence of block polymer soft templating (Figure 2A). Comparing PEO_428_-*b*-PHA_265_ and PEO_251_-*b*-PHA_95_, the former results not only in larger defined spherical mesopores with (37 ± 6) nm as pore diameter but also an increased order (Figure 2E). Such a difference in the mesoscopic ordering might be a consequence of both the block length and the polymer-to-precursor ratio, the origin of which will be addressed in a separate study varying the template concentrations. The ordered pore structures, together with uniform pore diameters visible in TEM micrographs, implement important mechanistic insight. This uniformity is possible only as polymer degradation and carbonization of the carbon matrix are decoupled. Cross-linking of the lignin-containing matrix at moderate temperatures yields a loose carbon framework through which removal of template degradation products in applied nitrogen gas flow is still possible.

Yet, further extension of the PHA block length yielded variable ellipsoidal pore shapes and a larger variation in diameters (Figure 2F). In general, as shown in Table 1, the larger the block copolymer chain lengths, the broader the distribution of hydrodynamic micelle diameters and pore sizes subsequently. Simultaneously to growing pore diameters, pore wall thicknesses of carbons with spherical pores were extended (Table 1). Evaluation of the SEM micrographs confirmed the trends determined by TEM regarding the pore size in relation to the respective block copolymers (Table S2). Notably, SEM images of the templated carbons display well-defined mesopores throughout the monolithic materials’ entirety, especially the absence of dense nontemplated carbon (Figure S10).

**Figure 3:**
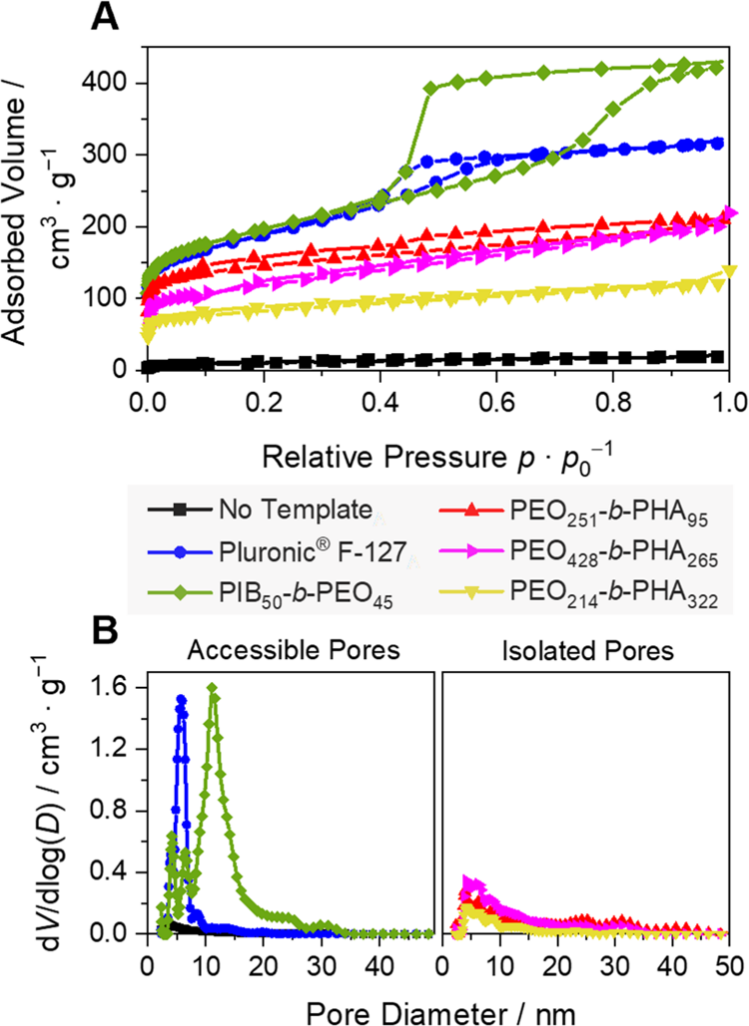
(A) Nitrogen isotherms of synthesized lignin-based carbon and (B) derived pore size distributions by applying a nonlocal density functional theory (NLDFT) kernel for carbon with cylindrical pore geometry to the adsorption branch.

To further gain knowledge of the pore morphology introduced by soft templating, nitrogen physisorption experiments (*T* = 77 K) were conducted (Figure 3). The lack of porosity in the nontemplated carbon sample, as indicated in the physisorption isotherm (Figure 3A) and pore size distribution (Figure 3B), agrees with TEM observations. Taking a closer look at the polymer-templated carbons, templating with Pluronic F-127 and PIB_50_-*b*-PEO_45_ led to isotherms with H2-type-shaped hysteresis loops^33^ and a pronounced nitrogen uptake (Figure 3A), indicating significant mesopore volume. To evaluate the desorption mechanism and whether pore blocking or cavitation network effect might cause delayed evaporation, especially for PIB_50_-*b*-PEO_45_-templated carbon, further investigation by varying the adsorptive or recording a series of hysteresis scans is necessary,^34^ which is not in the focus of the present study. Nevertheless, the occurrence of hysteresis is associated with restricted access to the pore cavity. The pore size distributions in Figure 3B were determined by applying an NLDFT kernel for carbon with a cylindrical mesopore geometry on the adsorption branch. Instead, using an NLDFT analysis assuming a spherical geometry for the large mesopores is expected to yield up to 50% larger pore diameters. These would present an even larger discrepancy with observed TEM results, which might be caused by the assumption of graphitic carbon with different polarities compared to the present nongraphitic, hard carbon implemented in the NLDFT kernel. Hence, an NLDFT kernel for cylindrical mesoporous carbon is applied, though being aware of pore diameters being slightly larger. It reveals global maxima at 6 nm (Pluronic F-127), respectively 11 nm (PIB_50_-*b*-PEO_45_), i.e., slightly larger pore diameters compared to mean pore diameters determined by averaged TEM measurements. Physisorption experiments of the carbon templated with PEO_*n*_-*b*-PHA_*m*_ polymers show different results. They display characteristic carbon microporosity with initial nitrogen uptake in the isotherm at a small relative pressure in all templated carbon materials. Yet, despite the abundance of mesopores displayed in TEM and SEM images and also contrary to SAXS analysis (see below), quite surprisingly, the overall pore volume is comparably small and hysteresis loops are absent in recorded isotherms (Figure 3A), thereby suggesting the presence of micropores and small mesopores as dominant pore populations, but no pores beyond approximately 10 nm. In agreement, derived pore size distributions reveal micro- and small mesopores but lack an indication of large mesopores of 20–50 nm (Figure 3B), suggesting that these voids are isolated from each other and inaccessible to nitrogen, which is astonishing in light of the quite high template-to-precursor ratio.

**Figure 4:**
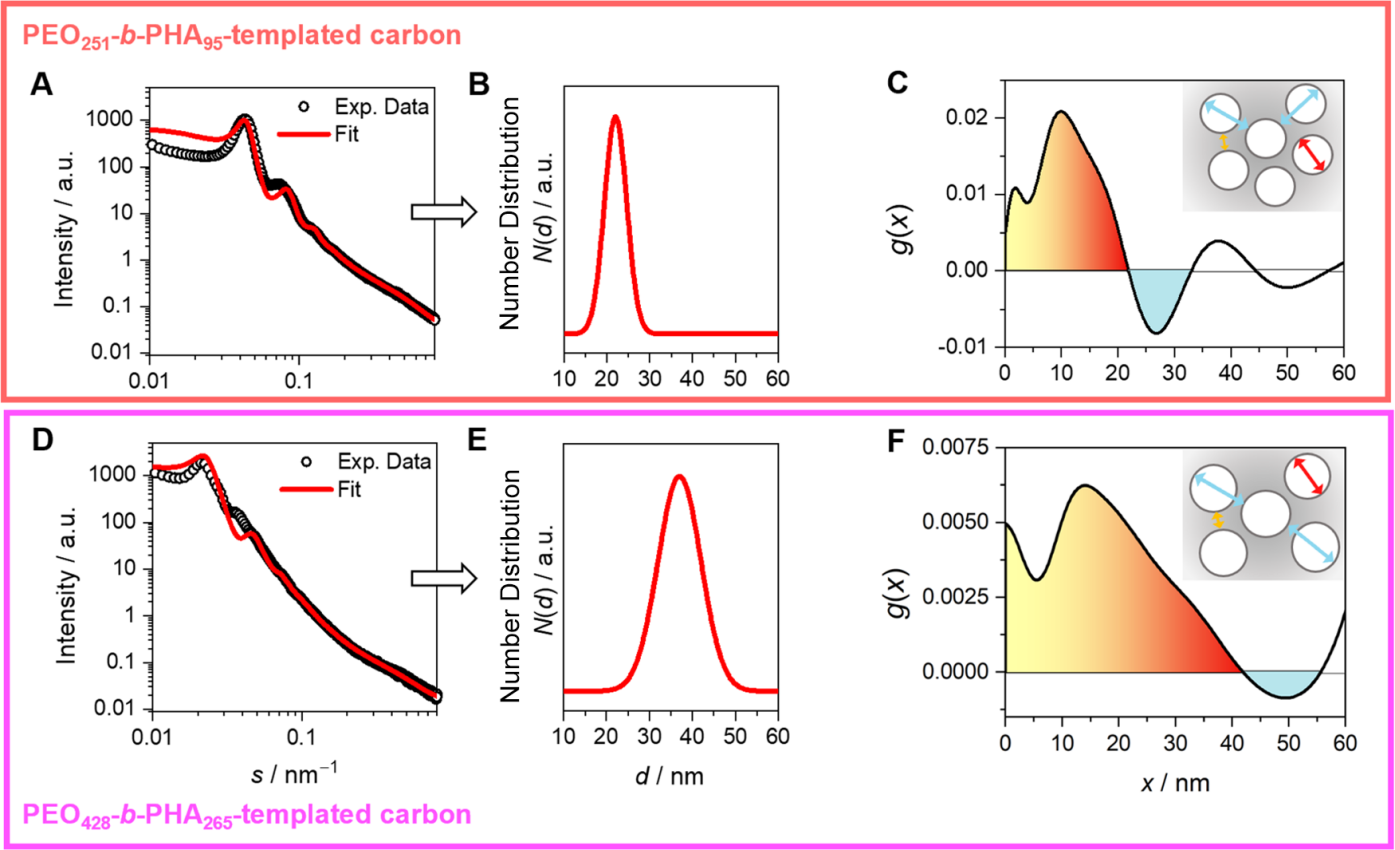
(A, D) Experimental SAXS data *I*(*s*) and fits (solid line) based on the Percus–Yevick approach with *s* = 2 sin(θ) λ^–1^, as well as (B, E) obtained number distributions of pore diameters induced by each respective template. Furthermore, chord length distributions obtained by converting experimental SAXS data fit for (C) PEO_251_-*b*-PHA_95_-templated and (F) PEO_428_-*b*-PHA_265_-templated mesoporous carbon. Contributions evoked by penetration of micropores and small mesopores (approximately <5 nm, yellow), pore walls (orange), and large templated mesopores (red), as well as pore-to-pore distances (cyan) are color-coded and sketched in a scheme. In the scheme, microporosity was omitted for the sake of clarity.

To address this apparent discrepancy between physisorption and SEM/TEM results for carbons templated with PEO_*n*_-*b*-PHA_*m*_ polymers, SAXS was utilized as a tool to detect all mesopores, both accessible and isolated ones. In the following, carbon templated with PEO_214_-*b*-PHA_322_ was disregarded due to its high polydispersity of pore sizes indicated by TEM and SEM; thus, focusing on carbons templated with PEO_251_-*b*-PHA_95_ and PEO_428_-*b*-PHA_265_. The SAXS patterns of both samples (Figure 4A and **D**) exhibit a series of distinct, broad maxima/oscillations, pointing to ordered arrays of mesopores. The first maximum at *s* = 0.043 nm^–1^ (PEO_251_-*b*-PHA_95_) and *s* = 0.021 nm^–1^ (PEO_428_-*b*-PHA_265_) can be interpreted as a “Bragg” reflection, *i.e*., caused by a defined average pore-to-pore distance. At larger *s* for both carbons, broad maxima/oscillations were observed, which might be interpreted as “Bragg” maxima or oscillations of the form factor of defined objects. Interestingly, an almost perfect Porod law is observed at larger *s* (*I*(*s*) ∝ 1/*s*^4^) (Figure S11C and S11F), confirming a well-defined two-phase system of larger mesopores/voids and the carbon matrix.

A quantitative SAXS evaluation of pore sizes upon scattering methods depends on the assumption of a geometrical model in order to fit the experimental data.^35^ As TEM micrographs (Figure 2) display spherical pores lacking a closed-packed arrangement with crystalline long-range order, a SAXS model on either a crystal-like array or on local order might be feasible. In the following, an approach was applied for the semiquantitative modeling of the SAXS data following the Percus–Yevick structure factor for hard spheres, as successfully applied previously.^36^ Aside from a hard-sphere potential, the Percus–Yevick structure factor presents a practical advantage by relying on only two parameters: the hard-sphere potential radius *R* and volume fraction η. Separated spherical pores thus fulfill a basic precondition of the Percus–Yevick approach, and our approach uses a polydisperse form factor for spheres.^32,36,37^ Modeling of the experimental SAXS data using this Percus–Yevick decoupling approach is shown in Figure 4A,4D. The overall shape of the SAXS curves is modeled quite well, although the position of the higher maxima cannot be achieved precisely. Yet, a reasonable pore diameter distribution was obtained for each mesoporous carbon material (Figure 4B,4E). The pore diameters of (22 ± 3) nm for PEO_251_-*b*-PHA_95_-templated carbon and (37 ± 5) nm for PEO_428_-*b*-PHA_265_-templated carbon are in good agreement with the TEM results. This SAXS analysis furthermore provides volume fractions of 44% for PEO_251_-*b*-PHA_95_-templated carbon and correspondingly 35% for PEO_428_-*b*-PHA_265_-templated carbon as well, which are in qualitative agreement with SEM/TEM analysis regarding the order of magnitude. Interestingly, the SAXS evaluation confirms a broader pore size distribution with an increasing block length of the applied polymer, in agreement with the TEM, SEM, and DLS analysis. In addition, we have modeled the SAXS patterns assuming a face-centered cubic (FCC) array of polydisperse spheres (see the SI file, Figure S12).^35^ It is seen that the position of the maxima can be adequately modeled as the higher-order reflections of the FCC packing, indicating a three-dimensional ordered packing, while it is not possible to fit the overall course of the curve. The average mesopore diameters obtained from this procedure and the Percus–Yevick-based approach agree reasonably well with each other for both materials (see Table S3). Yet, the wall thickness between these isolated mesopores cannot be calculated with acceptable accuracy, as the value of the average pore-to-pore diameter shows substantial uncertainty, being severely dependent on the packing model.

Thus, an alternative SAXS evaluation was performed that is not based on a distinct type of packing, namely, by the concept of chord length distributions (CLD). Since Porod′s law is valid for the SAXS data of both materials, corresponding to a two-phase system, the evaluation of the CLD is meaningful. Without any assumption of a geometrical model, CLD analysis presents a powerful tool to examine nanostructured materials with only local mesoscopic order.^26,38,39^ The CLDs evaluated by fitting the experimental SAXS data with analytical basic functions are displayed in Figure 4C (PEO_251_-*b*-PHA_95_-templated) and Figure 4F (PEO_428_-*b*-PHA_265_-templated).^40^ The parametrization process is demonstrated in the Supporting Information (SI File Figure S11). As a result of the absence of a sufficient amount of data points at very small scattering vectors *s*, causing a mathematical obstacle for the fitting procedure, artifacts appear in both CLDs at large radii (discussion provided in the SI, Figure S13). As a superposition of linear distances between phase boundaries, positive, respectively negative, contributions reflect the chord penetration of one, respectively two, interphases. Though this does not equal a pore size distribution, together with TEM as an independent method, it is justifiable to interpret the first positive contributions in both CLDs to be caused by varying pore diameters and wall thicknesses.^26,36,39^ According to Smarsly et al.,^36^ assigning the contributions of micropores and small mesopores (approximately <5 nm), pore walls, and large templated mesopores is even possible. In Figure 4C,4F, the respective contributions are color-coded in yellow, orange, and red.

As a main benefit, the CLD allows for the detection of small entities such as micropores and small mesopores.^36,39^ Compared to previously published block copolymer-templated material, the CLDs of the PEO_251_-*b*-PHA_95_- and PEO_428_-*b*-PHA_265_-based carbons exhibit only small contributions of entities below approximately 5 nm, i.e., attributable to small pores or thin pore walls. Regardless of this assignment, one can safely state that the materials contain a quite small concentration of micropores or mesopores between 2 and 5 nm (accessible or inaccessible). This finding can further explain the low degree of connectivity between the larger spherical mesopores. Large mesopore contributions in an *x*-range of approximately 18 to 22 nm for PEO_251_-*b*-PHA_95_-templated carbon and approximately 32 to 42 nm for PEO_428_-*b*-PHA_265_-templated carbon are in accordance with averaged pore diameter measurements in TEM micrographs. Reasonable values corresponding to TEM measurements are also obtained by the first minimum in the CLDs. For PEO_251_-*b*-PHA_95_-templated carbon, the sum of pore diameters and wall thickness determined by TEM corresponds to a pore-to-pore diameter of (28 ± 6) nm. Hence, the minimum in the CLD at 27 nm as the pore-to-pore distance is in good agreement. The CLD of carbon templated with PEO_428_-*b*-PHA_265_ with a minimum at 50 nm shows a greater deviation from TEM results of (44 ± 8) nm pore-to-pore diameter but is still within standard deviations.

Concluding pore morphology analysis, TEM, SEM, nitrogen physisorption, and SAXS analysis provide a coherent analysis, proving well-defined nanoscaled voids for all templates. In particular, PEO_*n*_-*b*-PHA_*m*_ polymers proved themselves as applicable templates, generating spherical mesopores of defined diameter and packing. Keeping a constant mass ratio of carbon precursor to template, PEO_*n*_-*b*-PHA_*m*_ polymers introduced isolated spherical pores in contrast to accessible pore morphologies by well-established Pluronic F-127 or PIB_50_-*b*-PEO_45_ templates as outlined with physisorption and SAXS experiments.

Next to the examination of the pore morphology, a thorough analysis of the carbon microstructure, *i.e*., of the graphenes and their stacking, is crucial in several ways as well. The dimension and disorder of the graphenes and their stacks (in the following termed “carbon microstructure”) might be as relevant for carbons applied in various energy material applications as the pore structure, especially for the transport of sodium in hard carbons. Here, the average interlayer spacing *a*_3_ presents a valuable parameter that is related to the (de)sodiation capability of sodium and is important for the elucidation of the mechanism of sodium storage in hard carbons, as elaborated in the recent literature.^23,41^ Moreover, a precise characterization of the carbon microstructure is a prerequisite to separate its impact from the impact of the pore morphology (diameter, shape, accessibility, and mutual connection) on electrochemical parameters. Also, investigating the general impact of introducing a template in combination with a carbonization process might reveal intriguing insights about the formation and reorganization of nanosized graphene sheets within nongraphitic carbons during thermal treatment. Therefore, microstructure elucidation by Raman spectroscopy and WAXS will be discussed, focusing on eight carbon samples produced with four different templates (nontemplated carbon, Pluronic F-127-templated carbon, PIB_50_-*b*-PEO_45_-templated carbon, and PEO_428_-*b*-PHA_265_-templated carbon) applying two different carbonization temperatures (900 and 1300 °C). The temperature of 1300 °C was selected as the upper choice to ensure that the conductivity behavior of the samples met the requirements as anode materials in a sodium-ion battery,^22^ which will be discussed in detail later in this study. Preservation of the mesopore structure upon the additional carbonization at 1300 °C as evaluated for the samples carbonized at 900 °C was confirmed by TEM (Figure S14), proving the thermal stability of the mesoporous scaffold.

**Figure 5:**
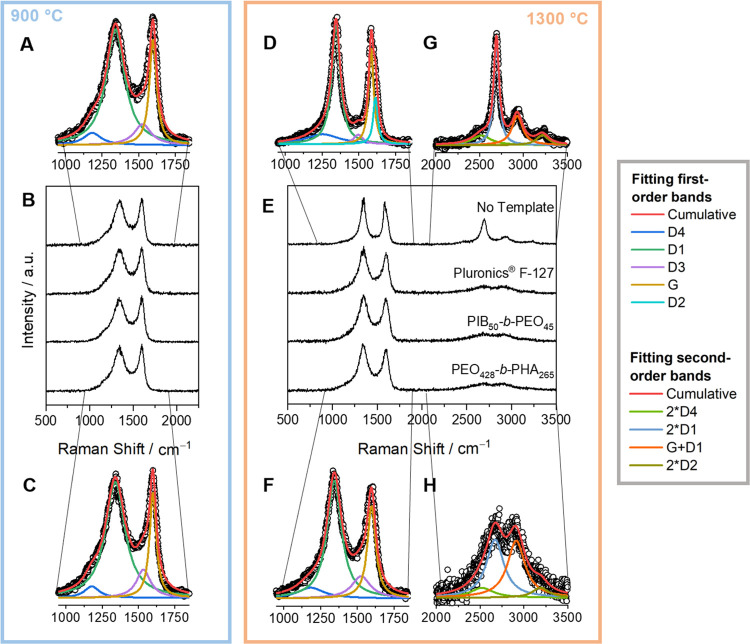
Raman spectra of (from top to bottom) nontemplated carbon, Pluronic F-127-templated carbon, PIB_50_-*b*-PEO_45_-templated carbon, PEO_428_-*b*-PHA_265_-templated carbon carbonized at (B) 900 °C and (E) 1300 °C. Fittings of first-order bands at 900 °C carbonized (A) nontemplated and (C) PEO_428_-*b*-PHA_265_-templated carbons. Likewise, fittings of nontemplated carbon first-order bands (D) and second-order bands (G), and PEO_428_-*b*-PHA_265_-templated carbon first-order bands (F) and second-order bands (H) after heat treatment at 1300 °C.

In Raman spectroscopy analysis, by applying an excitation wavelength of 532 nm, characteristic first-order bands in the spectral range of 1000 cm^–1^ to 1750 cm^–1^ and second-order bands from 2250 to 3500 cm^–1^ appear for all samples (Figure 5).^17,42−44^ All bands are broad and overlap as it is known for low carbonization temperatures and disordered nongraphitic structures.^17,43^ For spectral parameter elucidation, fitting the first-order bands includes all five reported Raman bands (commonly termed D1, D2, D3, D4, and G) applied with Lorentzian line shapes. Intensity maxima arise from the defect-activated band D1 appearing at ∼1350 cm^–1^ due to the A_1g_ symmetric vibrational mode of graphene edge defects and G band at ∼1585 cm^–1^ corresponding to a graphitic lattice mode with E_2g_ symmetry.^17,42,43^ Overlapping with the D3 band at ∼1500 cm^–1^ and D4 band at ∼1200 cm^–1^ appears in all recorded Raman spectra.^43,44^

For carbons carbonized at 900 °C, first-order band intensities and positions are qualitatively and quantitatively similar to the overview spectra (Figure 5B) and in-detail fittings in Figure 5A,5C. In contrast, differences among the samples appear at a carbonization temperature of 1300 °C. Whereas all templated carbon materials transform equally, as represented in Figure 5F, the first-order Raman spectra of the nontemplated carbon can be distinguished from all other samples upon such higher temperature treatment. With a pronounced asymmetric shape, the G mode overlaps with the D2 band appearing at ∼1620 cm^–1^, corresponding to the same graphitic lattice mode as the G band involving graphene layers at the surface (Figure 5D).^43^ As known in the literature, the D2 band appears upon higher carbonization temperatures, inducing a more ordered graphene lattice.^16,43,45^ Hence, notably, the appearance of the D2 band in nontemplated carbon carbonized at 1300 °C suggests qualitatively higher structural order compared to templated carbon. In addition, the increase in structural order is supported by the most pronounced decline of the full width at half-maximum (fwhm) within Raman modes of nontemplated lignin-derived carbon at 1300 °C,^43,17^ which is further underlined by consideration of the second-order bands. As Raman spectra of all samples carbonized at 900 °C resemble one another in the spectral range from 2250 to 3500 cm^–1^ (see Figures S15 and S16), combinations of graphitic lattice vibration modes and overtones appear different for nontemplated carbon treated at 1300 °C (Figure 5G) compared to all other templated samples (Figure 5H). Fitting the second-order modes was applied by considering 2*D1, 2*D2, and 2*D4 overtones next to the G+D1 combination band with Lorentzian profiles each.^17,43,44^ As the 2*D1 overtone, though splitting into two 2*D1_1_ and 2*D1_2_ peaks, appears in Raman spectra of graphite as well, the absence of or a low-intensity broad 2*D1 overtone points out a low degree of stacking within investigated templated carbon materials. In contrast, nontemplated carbon carbonized at 1300 °C exhibits a pronounced 2*D1 band shaped as a single Lorentzian, well-known for turbostratic nongraphitic carbon.^17,44,46^ Next to comparing templated and nontemplated lignin-derived carbon, an appealing comparison can be made with nontemplated lignin-phloroglucinol-based carbon (this study) and phenol-formaldehyde resin-based carbons (PF-R) reported by Schuepfer et al.^17^ For PF-R materials, the appearance of the 2*D1 band was first observed upon carbonization at 1800 °C. The presence of the aforementioned band at a significantly lower carbonization temperature of 1300 °C within investigated lignin-based carbons suggests an accelerated growth and increase in structural order of the graphenes compared to conventional resin-based carbons. In a qualitative comparison of Raman spectra alone, decreasing fwhm and increasing D2 and 2*D1 band intensity of nontemplated lignin-derived carbons are clear indicators of decreasing disorder and layer extension increase compared to all templated carbons.^16,17^ Hence, it can be stated already that introducing templates causes restriction in graphene layer (re)organization during thermal treatment. Also, all different polymers seem to affect and hinder graphitization to the same extent.

**Figure 6:**
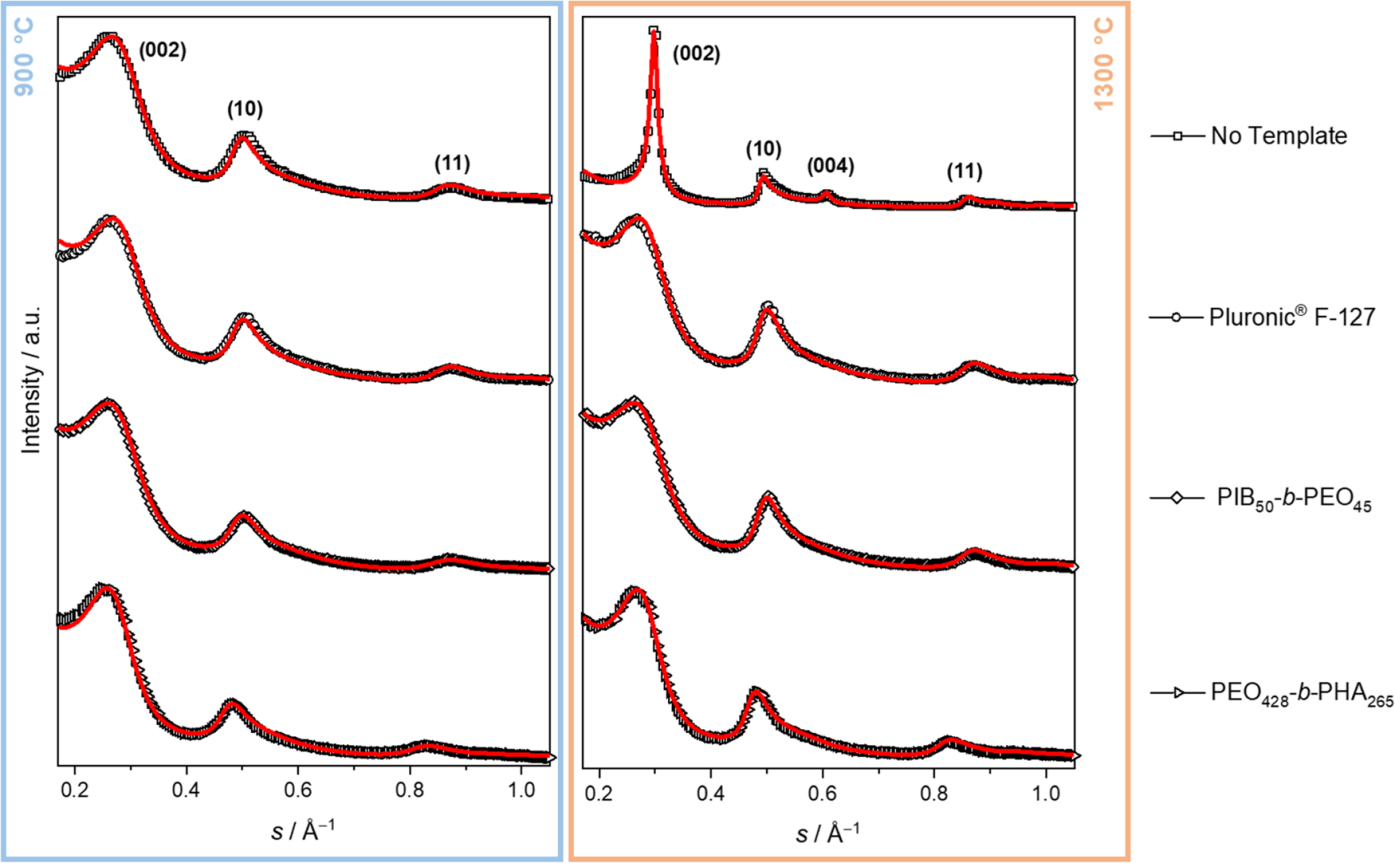
WAXS data of (from top to bottom) nontemplated carbon, Pluronic F-127-templated carbon, PIB_50_-*b*-PEO_45_-templated carbon, PEO_428_-*b*-PHA_265_-templated carbon, and fitting curves (solid line) obtained by the algorithm of Ruland and Smarsly^20^ as a function of the modulus of the scattering vector *s* = 2 sin(θ) λ^–1^.

For quantitative carbon microstructure analysis by Raman spectroscopy, several empirical relations determining the graphene extension *L*_a_ based on the intensity ratio of D1 and G band are described and applied in the literature.^47−50^ Though, as shown in extensive comparative studies by Schuepfer et al.,^17^ the commonly used Raman intensity ratio *I*_D_/*I*_G_ does not provide a universal correlation to determine *L*_a_. For instance, since the D band intensity depends on formed defects, whose type and density are related to precursor molecules and carbonization procedure, quantitative evaluation of Raman results ideally requires a combination with other methods determining the graphene extension *L*_a_.^16,17^ Several quantitative structural parameters can be obtained by wide-angle X-ray scattering (WAXS) analysis, which was applied to the carbon materials studied here. It is important to note that the overall class of nongraphitic carbons, to which hard carbons belong, shows turbostratic disorder in the graphene lattice and lacks crystallographic three-dimensional long-range order within the stacking of graphene layers, according to the IUPAC definition.^51^ An appropriate carbon microstructure evaluation based on the diffuse and overlapping (00*l*) and (*hk*) reflections of asymmetric shape in the obtained WAXS pattern (Figure 6) was possible by applying the algorithm developed by Ruland and Smarsly^20^ implemented in the *OctCarb* script developed by Osswald and Smarsly^21^ in combination with the GNU *Octave*(52) software. The approach by Ruland and Smarsly^20^ is based on fitting the experimental WAXS data over the whole angular range by a continuous model function obtaining up to 18 physical and geometrical parameters describing the carbon microstructure, especially in terms of dimension and disorder of the graphenes and their stacking. The theory of the WAXS algorithm is described in detail elsewhere.^18−21^ The obtained experimental WAXS data with corresponding fittings of the studied lignin-derived carbons carbonized at 900 and 1300 °C are demonstrated in Figure 6.

**Figure 7:**
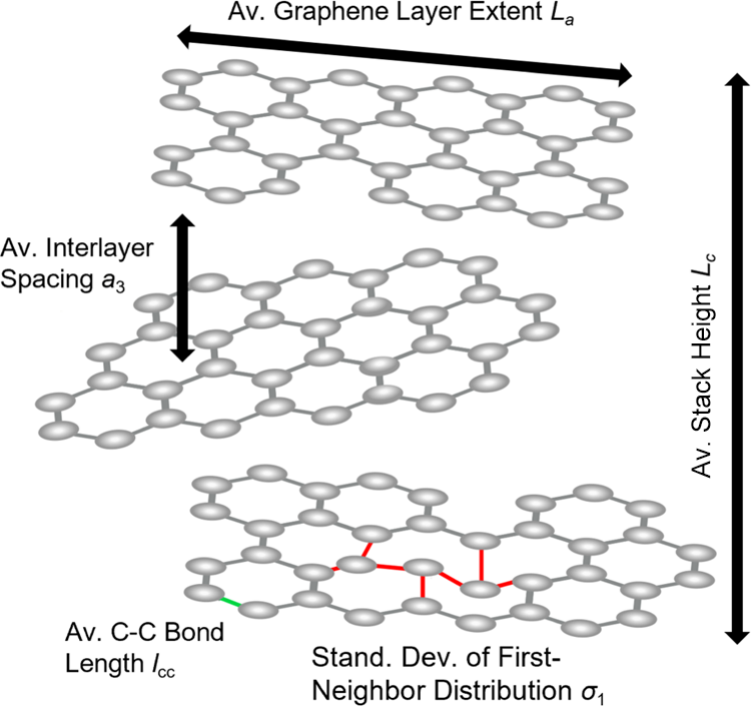
Schematic Illustration of most relevant layer and stacking parameters describing turbostratic nongraphitic carbon.

Already, the qualitative comparison of the WAXS patterns alone is in accordance with insights obtained by Raman spectroscopy: Broad reflections govern the experimental WAXS data for all investigated carbons, but the nontemplated carbon carbonized at 1300 °C (Figure 6, top right) presents a quite interesting exception. Here, significantly sharper reflections are obtained. Whereas the (004) reflection can only be perceived as asymmetry or shoulder in the vicinity of the (10) reflection in all other patterns due to a large stacking disorder, it appears defined and isolated in the WAXS data of 1300 °C carbonized and nontemplated lignin-derived carbon, indicating a higher order within the graphene layers. Going beyond such qualitative analysis, the fitting approach by Osswald and Smarsly^21^ was successfully applied to the WAXS data, achieving reasonable fitting over almost the entire experimental WAXS range. Hence, the structural parameters derived from them can be regarded as reliable. The most important obtained parameters regarding the size of graphene layers and stacking and respective disorder parameters are listed in Table 2 and schematically illustrated in Figure 7. Interestingly, the qualitative interpretation of Raman spectroscopy and WAXS data is supported by the determination of quantitative structure parameters based on the WAXS fitting approach.^19^

**Table 2:** Parameters Received by WAXS Data Fitting Applying the Algorithm by Ruland and Smarsly^20^

applied template			no template		pluronic F-127		PIB_50_-*b*-PEO_45_		PEO_428_-*b*-PHA_265_	
carbonization temperature			900 °C	1300 °C	900 °C	1300 °C	900 °C	1300 °C	900 °C	1300 °C
Layer Parameters										
	av. graphene layer extent	*L*_a_/Å	27.8	62.5	23.8	27.8	26.5	28.6	23.8	27.8
	av. C–C bond length	*l*_cc_/Å	1.408	1.414	1.405	1.406	1.409	1.406	1.408	1.409
	stand. dev. of first-neighbor distribution	σ_1_	0.13	0.07	0.12	0.11	0.17	0.12	0.15	0.12
Stacking Parameters^a^										
	av. stack height	*L*_c_/Å	∼10	32	∼10	∼10	∼10	∼10	∼10	∼10
	av. interlayer spacing	*a*_3_/Å	3.56	3.45	3.58	3.52	3.59	3.54	3.60	3.50
	stand. dev. of interlayer spacing	σ_3_/Å	0.53	0.25	0.58	0.40	0.47	0.39	0.40	0.29

a Except for the nontemplated carbon carbonized at 1300 °C, due to the absence of higher-order reflections, values of stacking parameters are to be considered with caution, as explained below.

Multiple comparisons can be drawn. First, an analysis of the layer-related parameters reveals that increasing the carbonization temperature leads to a higher degree of intralayer order within the carbon microstructure. This is evidenced by the enlarged average graphene layer extent (*L*_a_) and reduced disorder parameter (σ_1_) observed across all carbon materials if the two treatment temperatures are compared, i.e., 900 °C vs 1300 °C. A significantly lower increase in *L*_a_ (8–17%) was observed across all templated mesoporous carbon materials compared to a much larger growth in *L*_a_ (from 2.8 nm to approximately 6 nm, i.e., by 125%) for the nontemplated carbon. Similarly, σ_1_ decreased by 46% in nontemplated compared to 8–29% in templated carbon. Additionally, an extended average C–C bond length *l*_cc_ approaching the value of 1.421 Å in graphite^53^ is observed for nontemplated carbon, while the *l*_cc_ values of templated carbons are kept rather constant. Therefore, while the microstructures of all carbons carbonized at 900 °C are similar, further graphene growth and increasing order upon higher carbonization temperatures are significantly impeded when polymers are used as soft templates. Overall, this detailed analysis reveals the interesting insight that the templating of carbon has an impact on the microstructure, even when high-temperature post-treatments are carried out after template removal.

As mentioned before, not all WAXS reflections are resolved well due to the intrinsic properties of nongraphitic carbons and a substantial disorder. Yet, valid structure parameters depending on the signal width are only obtained by the applied evaluation technique if at least two reflections of the same lattice plane family caused by intralayer, respectively interlayer, scattering can be fitted precisely, i.e., for instance, the (10) and (20) reflections.^16^ Considering the (00*l*) reflections, this is not the case, as noted for the (004) reflection. Together with the finite measurement range of standard WAXS laboratory setups limiting the number of recorded reflections, this shows the limitations of possible microstructure evaluation within this study, causing the stacking height *L*_c_ only to be determined in terms of order of magnitude. Again, the nontemplated carbon sample carbonized at 1300 °C is an exception here due to more advanced structural ordering in the two-dimensional graphene layers and their stacking. Regarding the average interlayer spacing *a*_3_, the value is usually determined directly from the position of the (002) signal maximum by Bragg′s law. Such procedure, however, involves significant uncertainties and yields unrealistic values, *e.g*., ∼ 3.8 Å for templated carbons carbonized at 1300 °C (Table 3). By contrast, the WAXS fitting procedure takes into account effects possibly influencing the signal position and width, in particular the distribution of *a*_3_, but also small-angle scattering contributions, etc. The systematic overestimation of the interlayer spacing *a*_3_ of nongraphitic carbons applying Bragg′s law already justifies the comprehensive WAXS analysis applying the fitting procedure and algorithm by Ruland and Smarsly,^20^ especially as mechanistic studies of hard carbon anodes for sodium storage still rely on the interlayer spacing *a*_3_ determined by Bragg′s law.^23,41^ The better the fitting of the whole WAXS curve, the more reliable the values for *a*_3_ are received. The correlation between the interlayer spacing *a*_3_ of hard carbons in SIBs and the electrochemical performance will be discussed later.

**Table 3:** Overview of Structural Parameters of Nontemplated Carbon, Pluronic F-127-Templated Carbon, PIB_50_-b-PEO_45_-Templated Carbon, and PEO_428_-b-PHA_265_-Templated Carbon Carbonized at 1300 °C Applied as Anode Materials for Sodium-Ion Batteries

applied template	av. interlayer spacing *a*_3_/Å applying the algorithm of Ruland and Smarsly^20^	av. interlayer spacing *a*_3_/Å by Bragg′s law	av. graphene layer extent *L*_a_/Å	av. stack height *L*_c_/Å	mean carbon mesopore diameter (TEM)/nm^a^	*I*_D1_/*I*_G_	mesoporous carbon BET surface area/m^2^ g^–1^
no template	3.45	3.45	62.5	32		1.23	326
pluronic F-127	3.52	3.75	27.8	∼10	5 ± 1	1.27	496
PIB_50_-*b*-PEO_45_	3.54	3.88	28.6	∼10	9 ± 1	1.18	571
PEO_428_-*b*-PHA_265_	3.50	3.75	27.8	∼10	37 ± 6	1.28	145

a Obtained by averaging TEM measurements.

**Figure 8:**
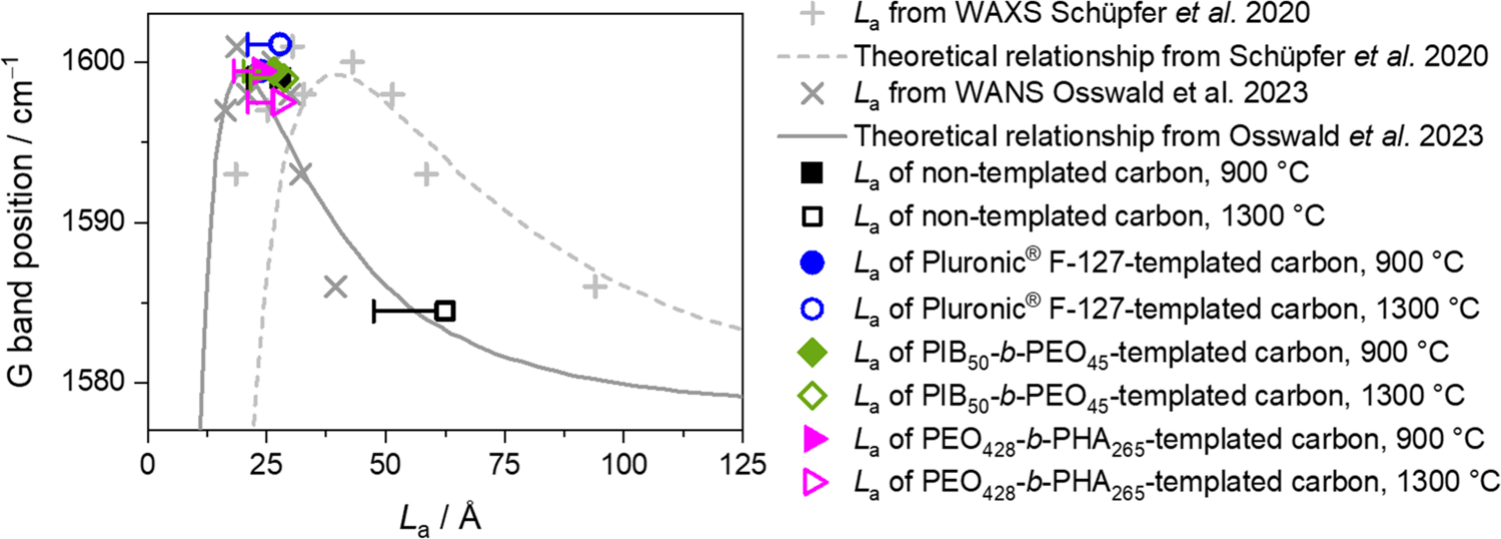
Relation of G band position to obtained graphene extension *L*_a_ acquired by WAXS (Schuepfer et al.^17^ and this study) and WANS (Osswald et al.^16^) of lignin-derived carbons (this study) and comparable PF-R carbons (Schuepfer et al.^17^ and Osswald et al.^16^). Reprinted or adapted with permission under a Creative Commons CC-BY 4.0 from ref (16) and ref (17). Copyright 2023 MDPI and 2020 Elsevier.

As an important advantage of this WAXS data evaluation, fitting of (10) and (11) intralayer reflections enabled a precise quantification of *L*_a_ and the parameter (σ_1_), which are meaningful parameters to describe the graphene layers. Thereby, the recently introduced advanced Raman spectroscopy analysis correlating spectroscopic quantities against WAXS analysis, introduced by Schuepfer et al.^17^ and elaborated by Osswald et al.,^16^ is applicable. Therein, the dependency of the G band position on the average layer extension *L*_a_ was discussed with empirical and theoretical relationships. As the G band position is nondispersive, comparing the Raman and WAXS analysis results in this study with the universal master curves of the above-mentioned literature^16,17^ (Figure 8) allows further assessment of the validity of microstructure analysis of lignin-based carbons.

The results of the current study are in good agreement with those of previous ones on comparable PF-R carbons, which belong to the class of hard carbons, too. It is worth mentioning that wide-angle neutron scattering (WANS) and the results applied by Osswald et al.^16^ provide a higher accuracy compared to WAXS analysis (Schuepfer et al.^17^ and this study) due to significant damping of WAXS data at larger *s* values caused by the atomic form factors, the impact of Compton scattering, and confined scattering range of *s* in typical Cu-Kα-WAXS laboratory instruments.^16^ Typically, *L*_a_ is overestimated, and σ_1_ shifted to higher values for such typical XRD setups.^16,54^ Pfaff et al.^54^ specified a maximum deviation of approximately 25% as the error range, determining *L*_a_ with standard laboratory WAXS setups. Considering this, corresponding error bars complement the obtained *L*_a_ values in Figure 8, representing microstructure analysis of the mesoporous lignin-derived carbons. Approaching the WANS-based master curve of Osswald et al.^16^ shows a good validness of the conducted microstructure analysis.

With the knowledge gained about the carbon microstructure, density measurements by helium pycnometry (Figure S17) can also be evaluated validly. First, a density value of 1.76 g cm^–3^ for the nontemplated carbon material treated at 900 °C is in accordance with other comparable hard carbons treated at similar carbonization temperatures known in the literature as in Badaczewski et al.^18^ or Kubota et al.^55^ For 900 °C as carbonization temperature, microstructure analysis revealed a comparable level of graphitization and a similar interlayer distance *a*_3_ for all samples, including the nontemplated carbon material. Hence, following the microstructure, templated carbons are expected to exhibit the same density value as the nontemplated carbon. However, a successive decrease in density is observed as templates are introduced with the lowest values for PEO_*n*_-*b*-PHA_*m*_-templated carbons. This can be explained by the extensive introduction of closed pores that are inaccessible to helium.^18,56^ The measured density is therefore not the density of the carbon skeleton only, but includes inaccessible pores. This further supports the existence of closed porosity most pronounced within the PEO_*n*_-*b*-PHA_*m*_-templated carbon materials, as pointed out with physisorption and SAXS analysis, already. For further discussion of density values, reference is made to the SI.

**Figure 9:**
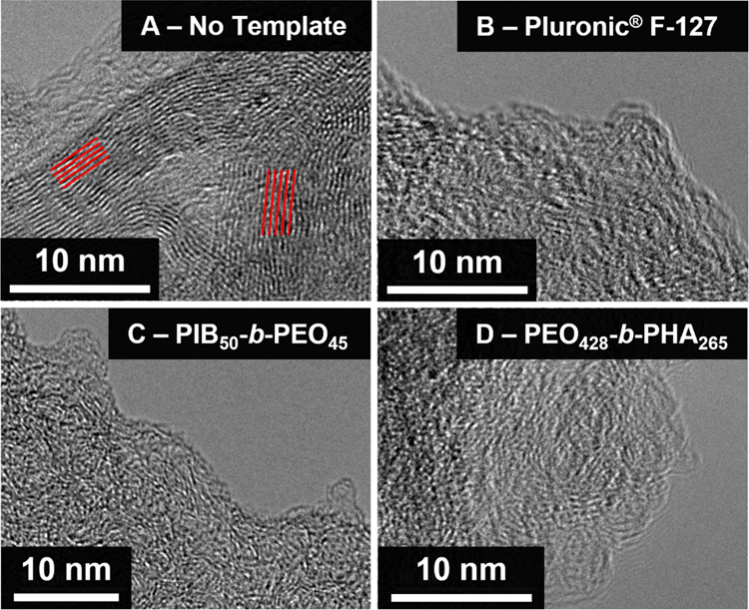
High-resolution TEM images of (A) nontemplated carbon, (B) Pluronic F-127-templated carbon, (C) PIB_50_-*b*-PEO_45_-templated carbon, and (D) PEO_428_-*b*-PHA_265_-templated carbon carbonized at 1300 °C. The pronounced level of graphitization and more ordered graphene layer organization is visible in the nontemplated carbon material and highlighted in red.

Concluding the microstructure analysis, the most pronounced level of graphene order is observed for the nontemplated carbon materials carbonized at 1300 °C and strongly underlined in Figure 9 (TEM) at first sight. Moreover, the complementary Raman and WAXS analysis demonstrates that all investigated mesoporous carbons resemble one another, especially upon applying the same carbonization temperature in their graphene structure, independent of the kind of polymer that was used as the soft template. These findings can be confirmed visually by high-resolution TEM images of nontemplated carbon, Pluronic F-127-templated carbon, PIB_50_-*b*-PEO_45_-templated carbon, and PEO_428_-*b*-PHA_265_-templated carbon carbonized at 1300 °C (Figure 9). In contrast to templated carbon materials, the nontemplated carbon sample exhibits aligned graphene layers after carbonization at 1300 °C, demonstrating a higher level of graphene order governing the carbon microstructure. Having addressed the influence of templating on the carbon transformation and, more importantly, confirmed the comparability of all mesoporous lignin-derived carbons in terms of their carbon microstructures, investigations of the pore morphology-induced effects in several energy material applications can take place.

As previously mentioned, nontemplated carbon revealed the highest degree of order of the graphene structure and the most compact microstructure, applying a carbonization temperature of 1300 °C. Meanwhile, the microstructure of all templated samples remains consistent at the same carbonization temperature, regardless of the templates used. The main distinguishing factor between these templated carbon materials is their mesopore size and structure, with some possessing interconnected pores/channels (PIB_50_-*b*-PEO_45_-templated and Pluronic F-127-templated carbon) and others exhibiting isolated closed pores (PEO_428_-*b*-PHA_265_-templated carbon). Finally, for characterization, we investigated the performance of four different carbon materials (nontemplated carbon, Pluronic F-127-templated carbon, PIB_50_-*b*-PEO_45_-templated carbon, and PEO_428_-*b*-PHA_265_-templated carbon, all carbonized at 1300 °C) as anode materials for sodium-ion batteries to help understand the sodium storage requirement regarding porosity and bulk phase. Therefore, an overview of the determined structure parameters for the respective carbons is given in Table 3. As discussed before, the intensity ratio of the D1 and G bands is still applied for quantitative structure analysis in the literature. Hence, this value is displayed in Table 3, as well. Although the nontemplated carbon exhibits a much larger level of “graphitization”, *I*_D1_/*I*_G_ values of templated carbons are similar. Again, this underlines that the determination of graphene layer extent *L*_a_ from the *I*_D1_/*I*_G_ value has to be interpreted with care, as demonstrated recently by Schuepfer et al.,^17^ which justifies our intensive state-of-the-art evaluation based on WAXS as complementary analysis.

**Figure 10:**
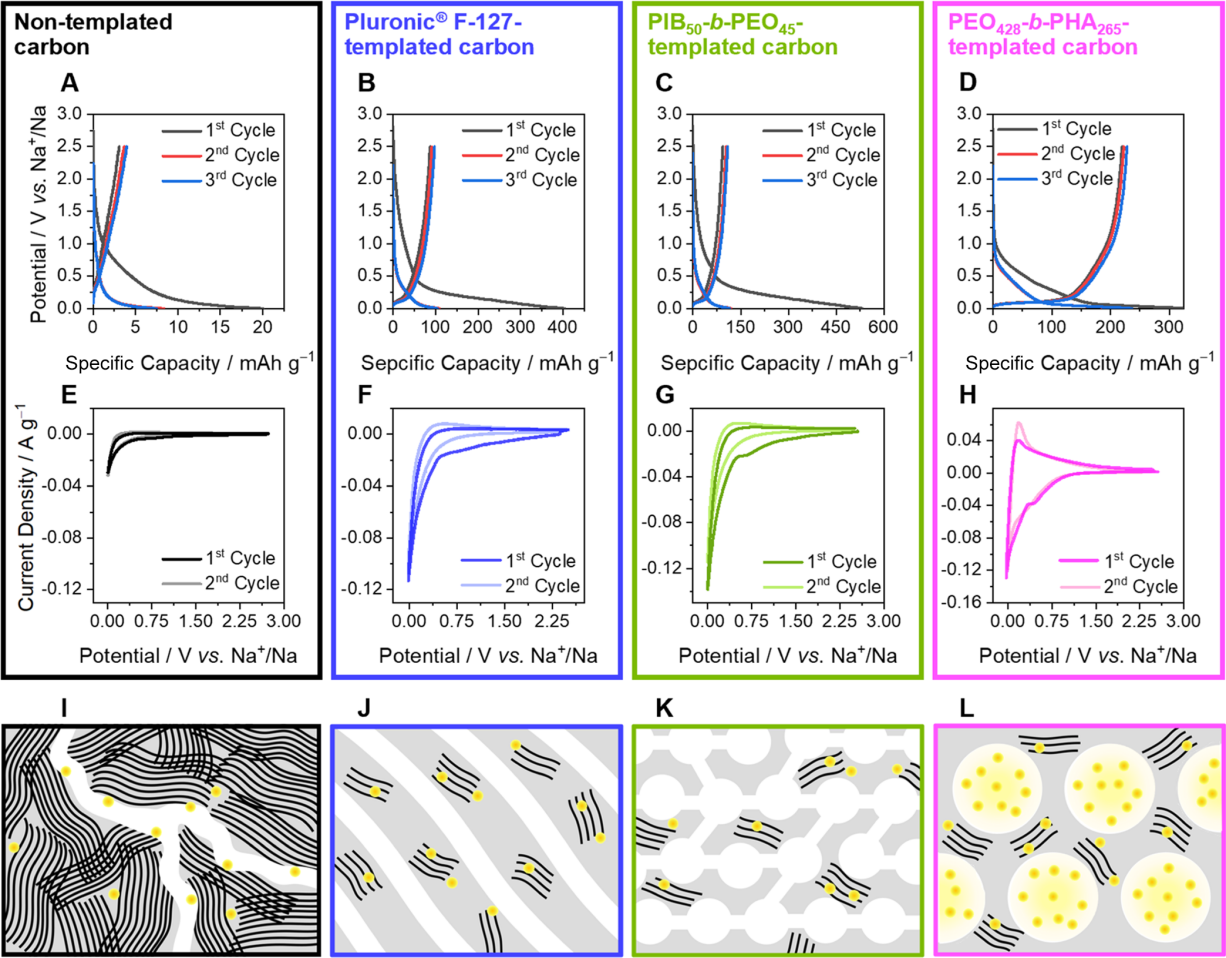
Discharge/charge profiles of sodium-ion batteries with (A) nontemplated carbon, (B) Pluronic F-127-templated carbon, (C) PIB_50_-*b*-PEO_45_-templated carbon, and (D) PEO_428_-*b*-PHA_265_-templated carbon as anode materials. The highest specific capacity with plateau capacity is observed only with PEO_428_-*b*-PHA_265_-templated carbon as anode materials with closed pores. Additionally, (E–H) cyclic voltammograms for the batteries with each respective anode material. Below, applied carbons in each sodium-ion battery are drawn schematically (I–L), illustrating carbon microstructure, which is comparable for all templated carbons, and the different pore morphologies. Interparticle voids, pore sizes, and pore connections do not have the claim to be represented true to scale.

The initial three charge–discharge profiles of all half cells at a current density of 30 mA g^–1^ are shown in Figure 10A–10D. As anticipated, the half cell with highly graphitized nontemplated carbon as anode material revealed negligible capacity due to the oriented graphite domain (schematically illustrated in Figure 10I) with the smallest average interlayer distances (Table 3), confirming the inability of sodium to form graphite intercalation compounds.^57^ Recent research into the mechanism of sodium-ion batteries has shown that different observed capacities can be attributed to sodium behavior. Specifically, capacities above approximately 0.1 V (sloping potential region) result from a combination of energetically different processes as well as the continuous structural distortion arising from the interlayer expansion that accompanies the insertion of sodium ions. As schematically illustrated in Figure 10I, small interlayer distances (Table 3) prevent sodium intercalation into the graphitic layers, causing very low capacity in the high-potential region already. Only sodium adsorption on the surface might be possible. For templated carbon materials, the interlayer spacing *a*_3_ is larger (Table 3), and as a result, a pronounced sloping capacity is observable (Figure 10B–10D). As the values of the interlayer spacing *a*_3_ and the yielded sloping capacity are comparable for all materials regardless of the applied template, the assignment of intercalation being responsible for the sloping capacity is reasonable and in accordance with the results of Anji Reddy et al.^58^ Enabled sodium intercalation for all templated carbon materials is illustrated in Figure 10J–10L. As mentioned previously, in comparison to the average interlayer spacing *a*_3_ determined by Bragg′s law, a consequent overestimation of *a*_3_ can be observed for the templated carbon materials. Only for nontemplated carbon do the values of interlayer spacing *a*_3_ determined by applying the algorithm of Ruland and Smarsly^20^ and Bragg′s law equal each other as the inaccuracy in determining *a*_3_ by Bragg′s law declines the more graphitic the carbon structure becomes. For templated nongraphitic carbon, comparison with the more accurately determined values by the algorithm of Ruland and Smarsly^20^ and resulting sloping capacities indicate that also smaller interlayer distances than the often stated 3.6 Å threshold distance^58^ allow sodium to intercalate. So far, experimental studies determining the threshold interlayer distance for sodium intercalation rely on the position of the (002) reflection and Bragg′s law.^23,41^ Here, our study raises attention to the fact that interlayer distances for nongraphitic carbons exhibiting broad reflections in WAXS experiments determined by Bragg′s law need to be handled with caution and eventually reevaluated.

In contrast to the sloping capacities, the plateau capacities in the low potential region differ from each other. For the closed pore PEO_428_-*b*-PHA_265_-templated carbon (Figure 10L), the half cell exhibited the highest specific capacity among all, with the plateau capacity observed exclusively here. As the comprehensive microstructure analysis revealed, only the pore morphology distinguishes the templated carbon materials, which permits justifying the difference in the plateau capacity with the porosity of the carbons. Agreeing with our observation here, the plateau capacities below 0.1 V are attributed to sodium filling into closed pores (Figure 10L) in the literature.^58,59^

**Figure 11:**
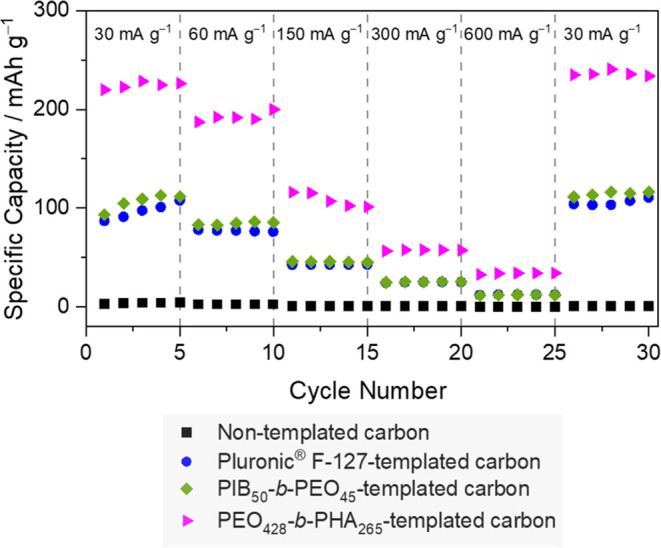
Rate performance of sodium-ion batteries with nontemplated carbon, Pluronic F-127-templated carbon, PIB_50_-*b*-PEO_45_-templated carbon, and PEO_428_-*b*-PHA_265_-templated carbon as anode materials at different current densities of 30, 60, 150, 300, and 600 mA g^–1^.

The pore filling by sodium is further supported by the peak observed at approximately 0.25 V in cyclic voltammetry, which is absent for Pluronic F-127-templated and PIB_50_-*b*-PEO_45_-templated carbon as the anode material (Figure 10E–10H). Also, the rate performances of all samples from 30 to 600 mA g^–1^ (Figure 11) are in accordance with the pore filling of Na^+^ exclusively within PEO_428_-*b*-PHA_265_-templated carbon with closed pores as anode material. The half cell with nontemplated carbon shows relatively low capacity mainly due to its more developed graphitic structure and narrow interlayer distance, as discussed previously. Among those templated carbons, PEO_428_-*b*-PHA_265_-templated carbon as the anode material shows the highest capacity in all different currents. The higher capacity is mainly attributed to the pore filling of Na^+^ from the plateau, which is not observed in other samples. Even after 900 cycles, the half cell with PEO_428_-*b*-PHA_265_-templated carbon exhibits the highest specific capacity in long-term cycling performance tests (Figure S18).

**Figure 12:**
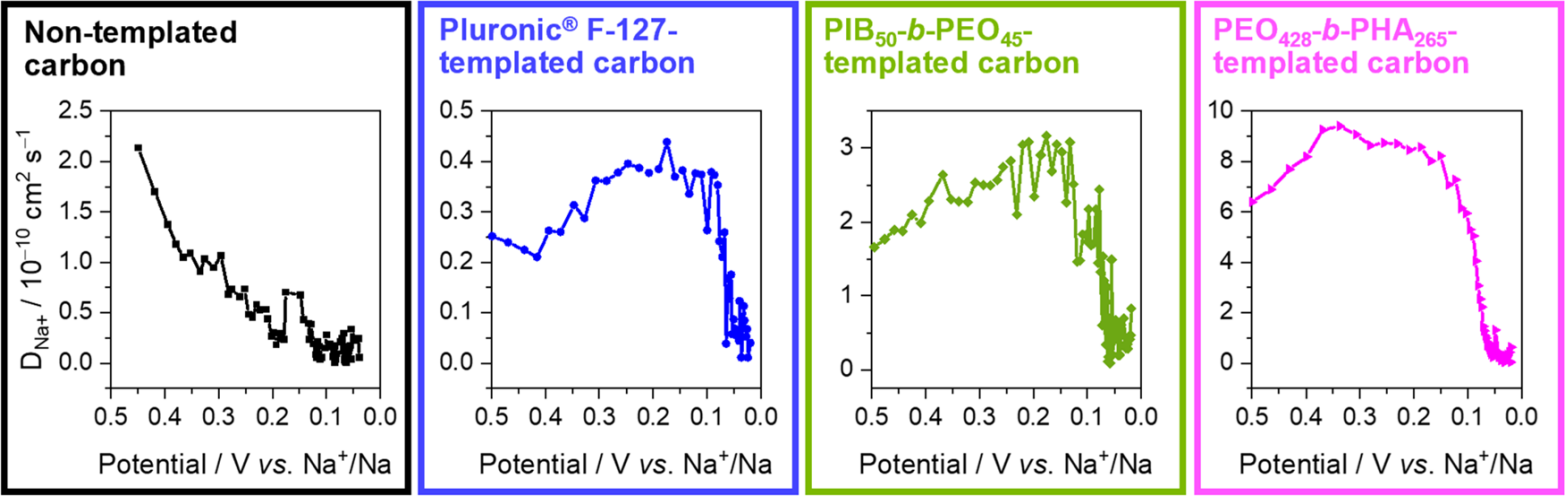
Na^+^ ion diffusion coefficients obtained from GITT tests for sodiation during the second cycle for sodium-ion batteries with nontemplated carbon, Pluronic F-127-templated carbon, PIB_50_-*b*-PEO_45_-templated carbon, and PEO_428_-*b*-PHA_265_-templated carbon as anode materials.

Figure 12 shows the calculated diffusion coefficients of Na^+^ (*D*_Na+_) in the hard carbon anode from the GITT tests for sodiation. For completeness, calculated *D*_Na+_ from GITT tests for desodiation can be found in the SI, Figure S19. It is worth noting that due to the intrinsic electrochemically active surface being different and difficult to measure, in this work, the electrode area was used in the GITT equation (see experimental methods). Thus, the absolute *D*_Na+_ may not be able to be compared between different samples, but it indicates a trend of *D*_Na+_ as a function of the potential. Together with the discharge/charge curve, the pore-filling mechanism was reported to be associated with an increase in *D*_Na+_ at the end of sodiation.^25,60,61^ From Figure 12, only with the PEO_428_-*b*-PHA_265_-templated carbon as the anode material is such a trend visible; hence, it is again reasonable to further conclude the Na^+^ pore-filling mechanism.

Additionally, half cells prepared with PEO_428_-*b*-PHA_265_-templated carbon material exhibited the highest initial Coulombic efficiency (ICE) of 67.7% while using Pluronic F-127-templated and PIB_50_-*b*-PEO_45_-templated carbon showed ICE values of 17.6 and 21.7%, respectively. This difference can be attributed to the surface area of the samples, where the higher surface area (Table 3) leads to the increased and excessive formation of undesired solid-electrolyte interphase (SEI) layers, resulting in decreased ICE. Therefore, the aim for rational anode material design is a high porosity consisting of pores being inaccessible for the electrolyte exclusively. In this way, porosity does not lead to an increased accessible surface area but contributes positively to enhanced electrochemical performances, as elucidated. In the future, accessibility of the pores should be further minimized while maintaining a high closed porosity, which could be attempted by adjusting the amount of polymer template, for instance. While introducing closed mesoporosity, another important parameter needs to be considered. Referring back to helium pycnometry density measurements (Figure S17), a lower density is a consequence as well, which can downgrade the volumetric capacity. Anyway, studies in the literature^62^ correlate a low density caused by introduced closed porosity with high plateau capacities of the respective half cell. Also, tested half cells with nontemplated carbon as anode materials showed only very poor performance, although exhibiting the highest density among the studied carbon materials. Hence, a correlation between a high density and good battery performance is not straightforward. Rather, a trade-off between a high density as a prerequisite for high volumetric capacities and maximized number of closed pores needs to be found for optimized anode materials.

Conclusively, with our comparative study applying different pore structures in hard carbon anode materials, the proposed mechanistic concept of sodium-ion storage by Au et al.^22^ could be supported, and the importance of in-detail carbon morphology and microstructure analysis toward the understanding and development of more efficient energy materials is underlined.

## Conclusion and Outlook

In summary, we demonstrated poly(ethylene oxide)-*b*-poly(hexyl acrylate) (PEO_*n*_-*b*-PHA_*m*_) block copolymers as suitable templates for the synthesis of lignin-derived mesoporous carbons featuring quite ordered arrays of spherical mesopores. Pore diameters of approximately 20–50 nm were feasible with the precise variation of the polymer block lengths *m*, mainly. Applying identical ratios between carbon precursors and templates PEO_*n*_-*b*-PHA_*m*_ block copolymers induced isolated spherical pores unlike well-established Pluronic F-127 or PIB_50_-*b*-PEO_45_ templates creating a smaller accessible channel or spherical pores, as proved by SAXS, SEM/TEM, and physisorption analysis. In future studies, systematically reducing the amount of templates might optimize the isolated character of pores regardless of the applied template and enable more efficient use of the polymer templates. Furthermore, in-depth microstructure evaluation with Raman spectroscopy and an advanced WAXS analysis of lignin-derived carbons was carried out to elucidate the transformation of the lignin-based precursor into graphene stacks. Notably, we find that the average interlayer spacing *a*_3_, obtained by the advanced WAXS data fitting approach, is smaller than the values determined by simple single-peak analysis (Scherrer analysis), which can serve as an important finding with respect to theoretical models for (de)sodiation in hard carbons. Regardless of the type of block copolymer applied as a template, surprisingly, templated mesoporous carbons exhibited a comparable microstructure in terms of evaluated graphene layer and stacking parameters after carbonization at the same temperature. Comparison to nontemplated carbon revealed that the usage of different soft templates hampers the graphitization process upon heat treatment significantly. The in-depth analysis of the carbon microstructure thus allowed us to separate the influence of pore morphology in synthesized nongraphitic carbons applied in sodium-ion batteries. Due to the feasible tunability of the pore size and their accessibility within our carbon materials, we created a series of excellent model materials for various electrochemical applications. Our preliminary results on half-cells, testing for applicability in sodium-ion batteries, reveal that PEO_428_-*b*-PHA_265_-templated carbon with closed mesopores as anode material showed an exclusive plateau capacity next to the sloping capacity in charge/discharge profiles. Open pore structures within the anode material exhibited a sloping capacity solely, causing significantly lower initial Coulombic efficiencies. In future studies, varying pore diameters in closed pore structures will enlighten the ideal pore size for an enlarged plateau capacity, causing enhanced sodium-ion battery performances. As the importance of carbon analysis for more effective energy materials was pointed out, further fundamental investigations might be initiated to enhance a detailed understanding of carbonization reactions of lignin-based carbons as well as the interaction and impact of different templates. Concluding, our synthesis of carbons with large mesopore sizes based on biomass-derived precursors opens great possibilities to enhance sustainability in various energy materials applications like electrocatalysis or energy storage.

## Supporting Information Available

Overview of applied amounts of reactants in polymer synthesis; ^1^H NMR spectra of all macroinitiators and PEO_*n*_-*b*-PHA_*m*_ block copolymers; GPC RI traces of two macroinitiators and all PEO_*n*_-*b*-PHA_*m*_ block copolymers; DLS data of Pluronic F-127, PIB_50_-*b*-PEO_45_, and PEO_*n*_-*b*-PHA_*m*_ templates in methanol; detailed GITT curve for calculation; comparison of templated pore sizes determined by TEM and SEM; SEM micrographs of all synthesized carbons carbonized at 900 °C; demonstration of SAXS data fitting for the CLD evaluation method and presence of Porod-law behavior; SAXS data modeling assuming a face-centered cubic (FCC) array of polydisperse spheres; comparison of obtained mean mesopore diameters applying different SAXS fitting models; CLD artifact discussion; TEM images of Pluronic F-127-templated, PIB_50_-*b*-PEO_45_-templated, and PEO_428_-*b*-PHA_265_*-*templated carbons carbonized at 1300 °C; complete Raman spectra of nontemplated, Pluronic F-127-templated, PIB_50_-*b*-PEO_45_-templated, and PEO_428_-*b*-PHA_265_*-*templated carbon carbonized at 900 °C; additional fitting of first- and second-order Raman bands of Pluronic F-127-templated and PIB_50_-*b*-PEO_45_-templated carbon; densities by helium pycnometry measurements of Pluronic F-127-templated, PIB_50_-*b*-PEO_45_-templated, and PEO_*n*_-*b*-PHA_*m*_*-*templated carbon carbonized at 900 °C; long-term cycling performance of sodium-ion batteries with templated carbons as anode materials; Na^+^ ion diffusion coefficients from GITT for desodiation during cycling of sodium-ion batteries with templated carbons as anode materials (PDF)
