# Supplementary material for: Lignin-Derived Mesoporous Carbon for Sodium-Ion Batteries: Block Copolymer Soft Templating and Carbon Microstructure Analysis
Source: Chem Mater. 2023 Dec 6;35(24):10416–33. doi: 10.1021/acs.chemmater.3c01520 (PMC10753804; doi:10.1021/acs.chemmater.3c01520)
Supplement: Supplementary file 1 — cm3c01520_si_001.pdf [file cm3c01520_si_001.pdf]

Lignin-derived Mesoporous Carbon for Sodium-Ion Batteries: Block Copolymer Soft Templating and Carbon Microstructure Analysis

Chantal Glatthaar[a,b], Mengnan Wang[b], Lysander Q. Wagner[a,c], Frederik Breckwoldt[a], Zhenyu Guo[b], Kaitian Zheng[b,d], Manfred Kriechbaum[e], Heinz Amenitsch[e], Maria-Magdalena Titirici[b,f]*, and Bernd M. Smarsly[a,c]**

a. Institute of Physical Chemistry, Justus-Liebig University, Heinrich-Buff-Ring 17, D-35392 Giessen, Germany

b. Department of Chemical Engineering, South Kensington Campus, Imperial College London, SW7 2AZ London, UK

c. Center of Materials Research, Justus-Liebig University, Heinrich-Buff-Ring 16, D-35392 Giessen, Germany

d. Chemical Engineering Research Center, State Key Laboratory of Chemical Engineering, School of Chemical Engineering and Technology, Tianjin University, Tianjin 300072, China

e. Institute of Inorganic Chemistry, Graz University of Technology, Stremayrgasse 9, A-8010 Graz, Austria

f. Tohoku University Advanced Institute for Materials Research (AIMR) Chome-1-1 Katahira, Aoba Ward, Sendai, Miyagi 980-0812, Japan

Supporting Information

Table S1. Overview of applied amounts of reactants in the SARA ATRP yielding PEO_n-*b*-PHA_m block copolymers.

Polymer	PEO_n-Br	Hexyl acrylate	TPMA	$CuBr_2$
PEO_{251}-*b*-PHA_{95}	2001 mg	3.37 mL	175 mg	67 mg
PEO_{428}-*b*-PHA_{265}	1999 mg	5.60 mL	88 mg	35 mg
PEO_{214}-*b*-PHA_{322}	1000 mg	6.97 mL	86 mg	22 mg

Syntheses of the PEO_n-Br macroinitiators were carried out using poly(ethylene oxide)methyl ether (PEO-OH, nominal average molecular weight of 10 kDa resp. 20 kDa, as provided by *Sigma-Aldrich*), 2-bromopropionic acid (99%, *Acros Organics*), 4-dimethylaminopyridine (DMAP, ≥99%, *Sigma-Aldrich*), and N,N'-dicyclohexylcarbodiimide (DCC, 99%, *Sigma-Aldrich*) in molar equivalents of 1 : 1.2 : 0.4 : 2.3 in anhydrous dichloromethane (DCM, 99.8%, *Acros Organics*). The PEO_n-Br macroinitiators were obtained with PEO block lengths of $n = 214$, 251, and 428 (regarding the average degree of polymerization as determined by end-group analysis (**Figure S1**, **S3**, and **S5**). In the following subsequent supplemental activator reducing agent atom transfer radical polymerization (SARA ATRP), anhydrous dimethylformamide (DMF, 99.8%, *Acros Organics*) served as solvent. **Table S1** displays the applied amounts of the respective macroinitiator PEO_n-Br, tris(2-pyridylmethyl)amine (TPMA, >98.0%, *TCI CO.*), $CuBr_2$ (>99%, water free, *Acros Organics*), and hexyl acrylate (98%, *Sigma-Aldrich*) as reactants yielding PEO_n-*b*-PHA_m copolymers of different block lengths. Detailed synthesis procedures can be found in recent literature.[1]

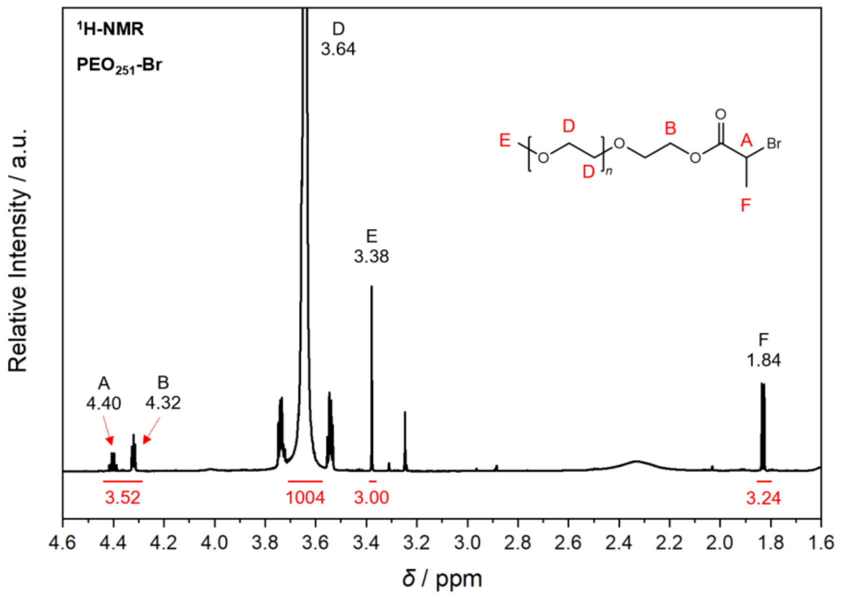

Figure S1. ^{1}H-NMR (700 MHz) spectrum of the PEO-Br macroinitiator (10 kDa), which was used for the synthesis of the PEO_{251}-*b*-PHA_{95} block copolymer, in $CDCl_3$. Peak assignments are in accordance with the displayed molecular structure.

The degree of polymerization was determined according to Wagner *et al.*[1] Integrals in the ^{1}H-NMR spectrum of the macroinitiator were used to determine the PEO block length *n*. Comparing intensity of signal D from the repeating unit with the head-group´s signal E intensity enables block length *n* determination as in equation (S1).

$$n = \frac{3\,I_D}{4\,I_E} \qquad \text{(S1)}$$

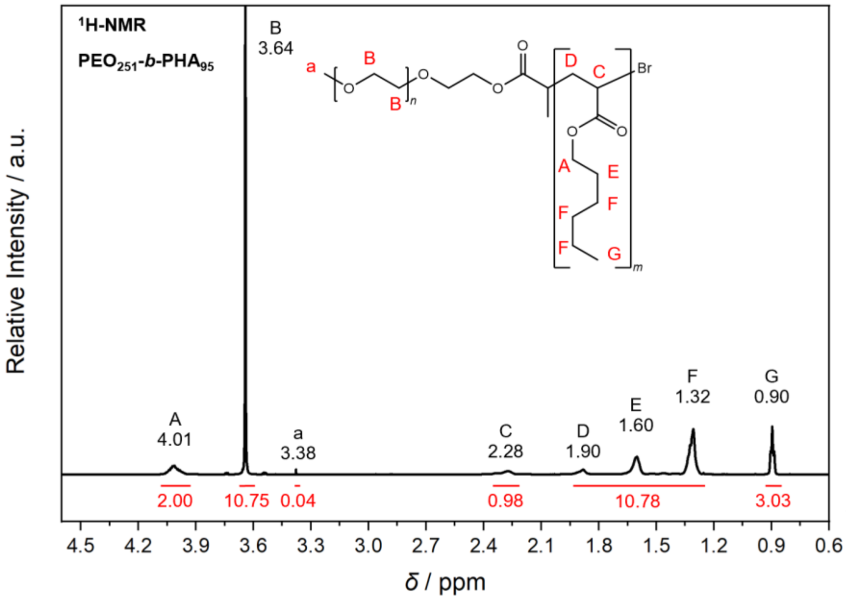

Figure S2. ^{1}H-NMR (700 MHz) spectrum of PEO_{251}-*b*-PHA_{95} block copolymer in $CDCl_3$. Peak assignments are in accordance with the displayed molecular structure.

Integrals in the ^{1}H-NMR spectrum of the respective block copolymer signals were used to determine the PHA block length *m*. As the PEO block length *n* was determined already, *m* could be calculated according to equation (S2).

$$m = \frac{4\,n}{I_B} \cdot \frac{\frac{I_A}{2} + \frac{I_C}{1} + \frac{I_D + I_E + I_F}{10} + \frac{I_G}{3}}{4} \qquad \text{(S2)}$$

For more detailed information about the evaluation of the degree of polymerization please refer to mentioned literature.[1]

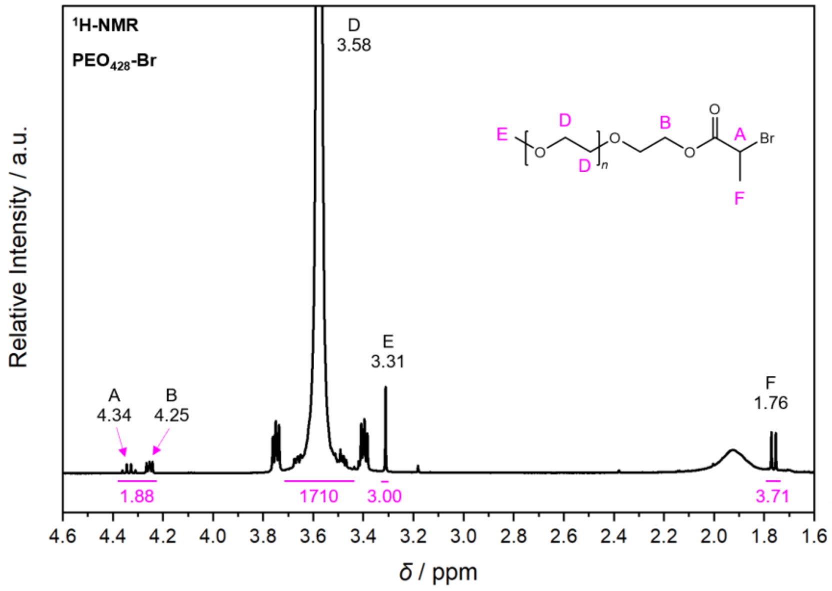

Figure S3. ^{1}H-NMR (400 MHz) spectrum of the PEO-Br macroinitiator (20 kDa), which was used for the synthesis of the PEO_{428}-*b*-PHA_{265} block copolymer, in $CDCl_3$. Peak assignments are in accordance with the displayed molecular structure.

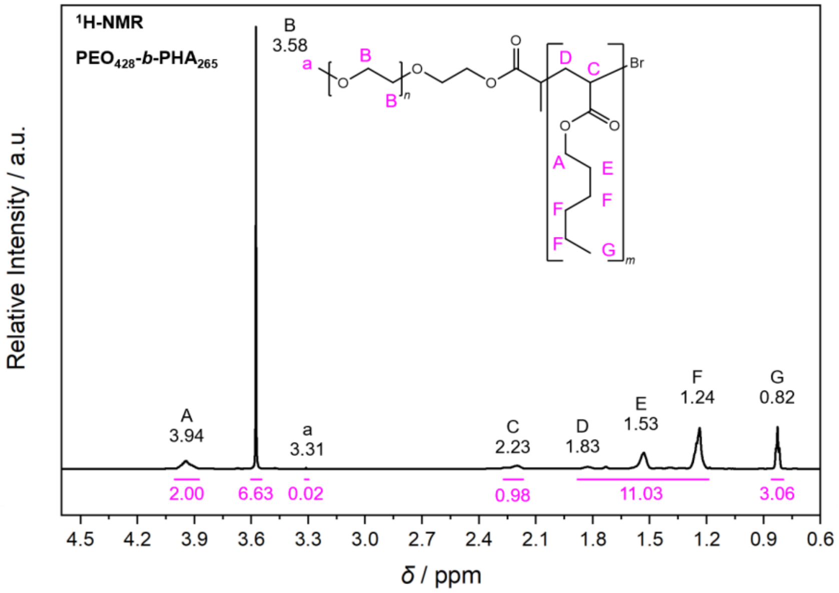

Figure S4. ^{1}H-NMR (700 MHz) spectrum of PEO_{428}-*b*-PHA_{265} block copolymer in $CDCl_3$. Peak assignments are in accordance with the displayed molecular structure.

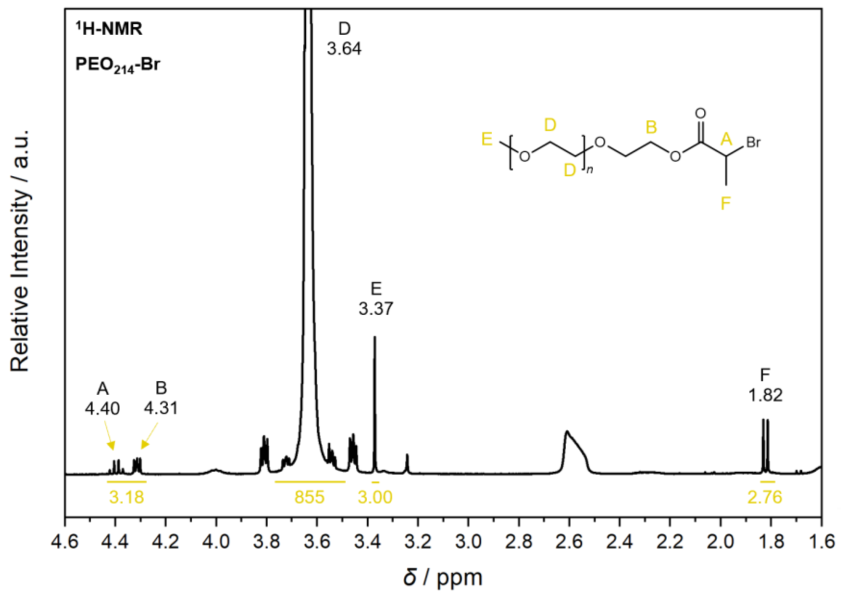

Figure S5. ^{1}H-NMR (400 MHz) spectrum of the PEO-Br macroinitiator (10 kDa), which was used for the synthesis of the PEO_{214}-*b*-PHA_{322} block copolymer, in $CDCl_3$. Peak assignments are in accordance with the displayed molecular structure.

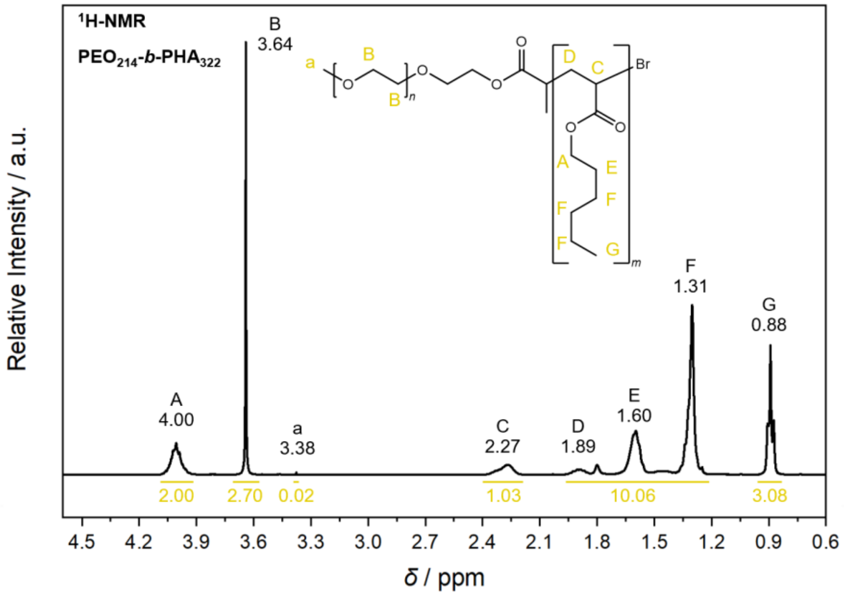

Figure S6. ^{1}H-NMR (400 MHz) spectrum of PEO_{214}-*b*-PHA_{322} block copolymer in $CDCl_3$. Peak assignments are in accordance with the displayed molecular structure.

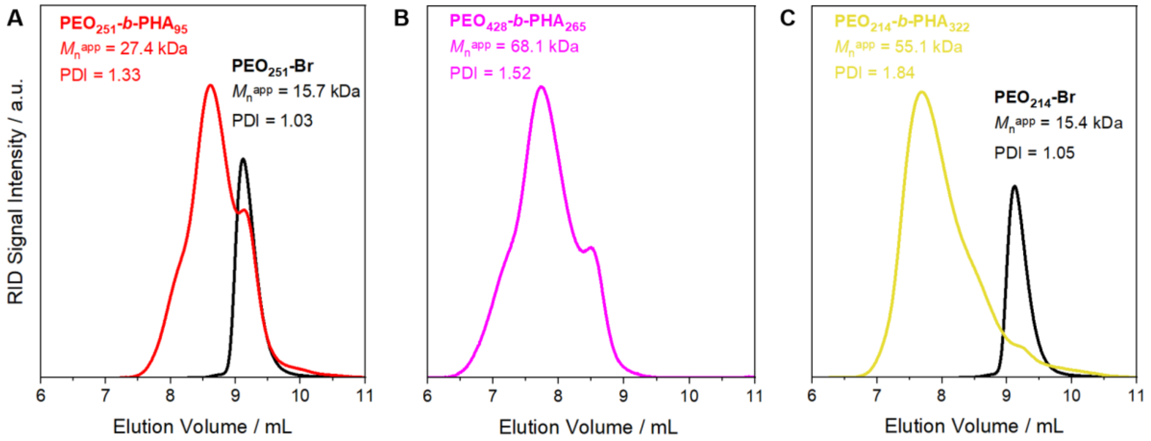

Figure S7. GPS curves from (A) PEO_{251}-*b*-PHA_{95} and PEO_{251}-Br macroinitiator, (B) PEO_{428}-*b*-PHA_{265}, and (C) PEO_{214}-*b*-PHA_{322} and PEO_{214}-Br macroinitiator. THF was used as eluent. For the PEO_{428}-Br macroinitiator no GPC measurements were carried out.

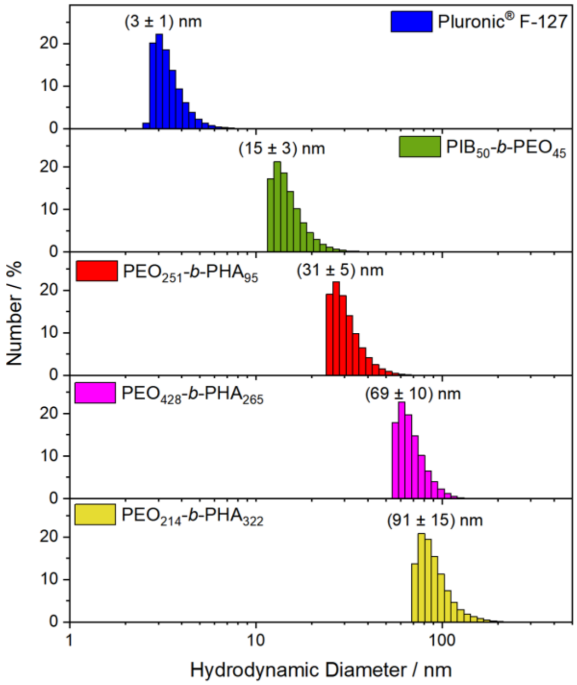

Figure S8. DLS data of (from top to bottom) Pluronic® F-127, PIB_{50}-*b*-PEO_{45}, and PEO_n-*b*-PHA_m block copolymers in methanol.

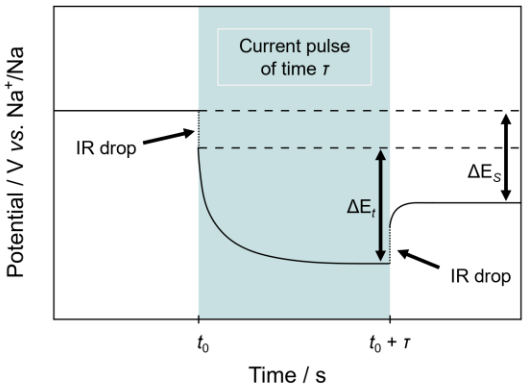

Figure S9. The detailed GITT curve for calculation.

Table S2. Comparison of pore size in resulting mesoporous carbon determined by TEM resp. SEM micrographs evaluation.

Template	Pluronic® F-127	PIB_{50}-*b*-PEO_{45}	PEO_{251}-*b*-PHA_{95}	PEO_{428}-*b*-PHA_{265}	PEO_{214}-*b*-PHA_{322}
Mean Carbon Mesopore Diameter (TEM) / nm[a]	5 ± 1	9 ± 1	23 ± 4	37 ± 6	52 ± 22
Mean Carbon Mesopore Diameter (SEM) / nm[a]	-	9 ± 2	21 ± 4	37 ± 4	58 ± 27

[a]Obtained by averaging TEM resp. SEM measurements

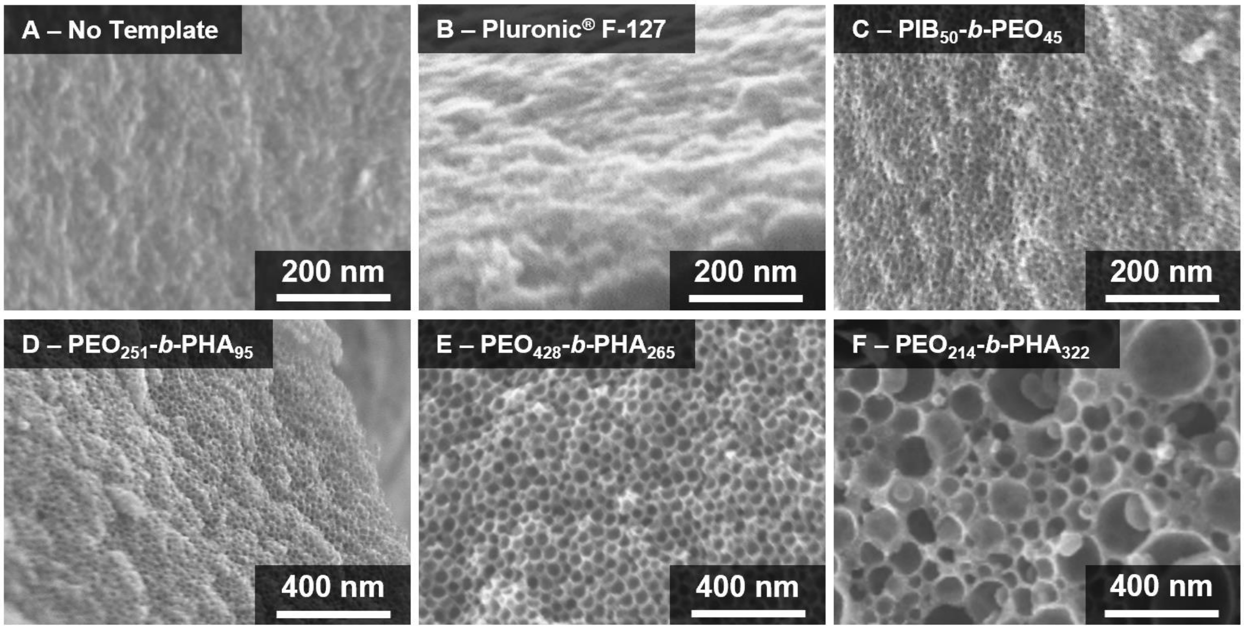

Figure S10. SEM micrographs of produced carbons after carbonization at 900 °C synthesized (A) without any template, (B) with Pluronic® F-127, and (C) with PIB_{50}-*b*-PEO_{45} as template. PEO_n-*b*-PHA_m polymers with varying block lengths (D) $n = 251$ and $m = 95$, (E) $n = 428$ and $m = 265$, and (F) $n = 214$ and $m = 322$ served as templates, additionally.

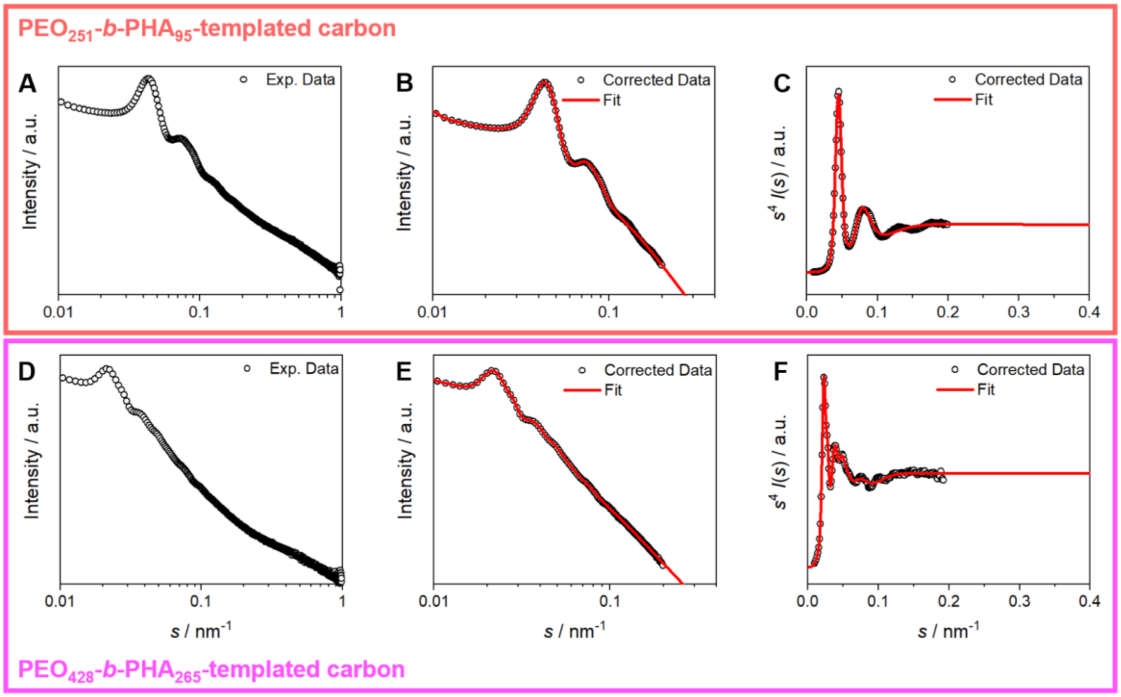

Figure S11. Parameterization process of SAXS data. Original experimental SAXS data of each respective carbon material (A and D) was fitted after background correction (B and E). Plotting s^4 $I(s)$ *vs*. scattering vector $s = 2\ \sin(\theta)\ \lambda^{-1}$ shows Porod-law behavior as data points fluctuate around a plateau as Porod constant.[2]

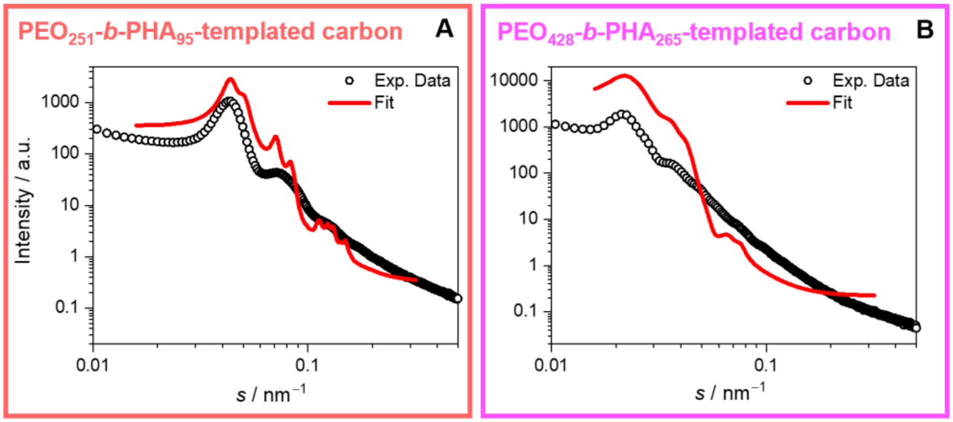

Figure S12. Experimental SAXS intensities $I(s)$ and fits (solid line) modelled assuming a face-centred cubic (FCC) array of polydisperse spheres (see Thomas *et al.*[3]) with $s = 2\sin(\theta)\,\lambda^{-1}$ of (A) PEO_{251}-*b*-PHA_{95}-templated and (B) PEO_{428}-*b*-PHA_{265}-templated mesoporous carbon.

Table S3. Comparison of obtained mean mesopore diameters applying the Percus-Yevick approach[4–7] or assuming a face-centered cubic (FCC) array of polydisperse spheres[3] for SAXS data modelling.

	Applied Packing Model for SAXS Fitting	PEO_{251}-*b*-PHA_{95}-templated carbon	PEO_{428}-*b*-PHA_{265}-templated carbon
Pore Diameter / nm	Percus-Yevick approach for polydisperse hard spheres	22 ± 3	37 ± 5
	FCC array of polydisperse spheres	21 ± 6	33 – 39

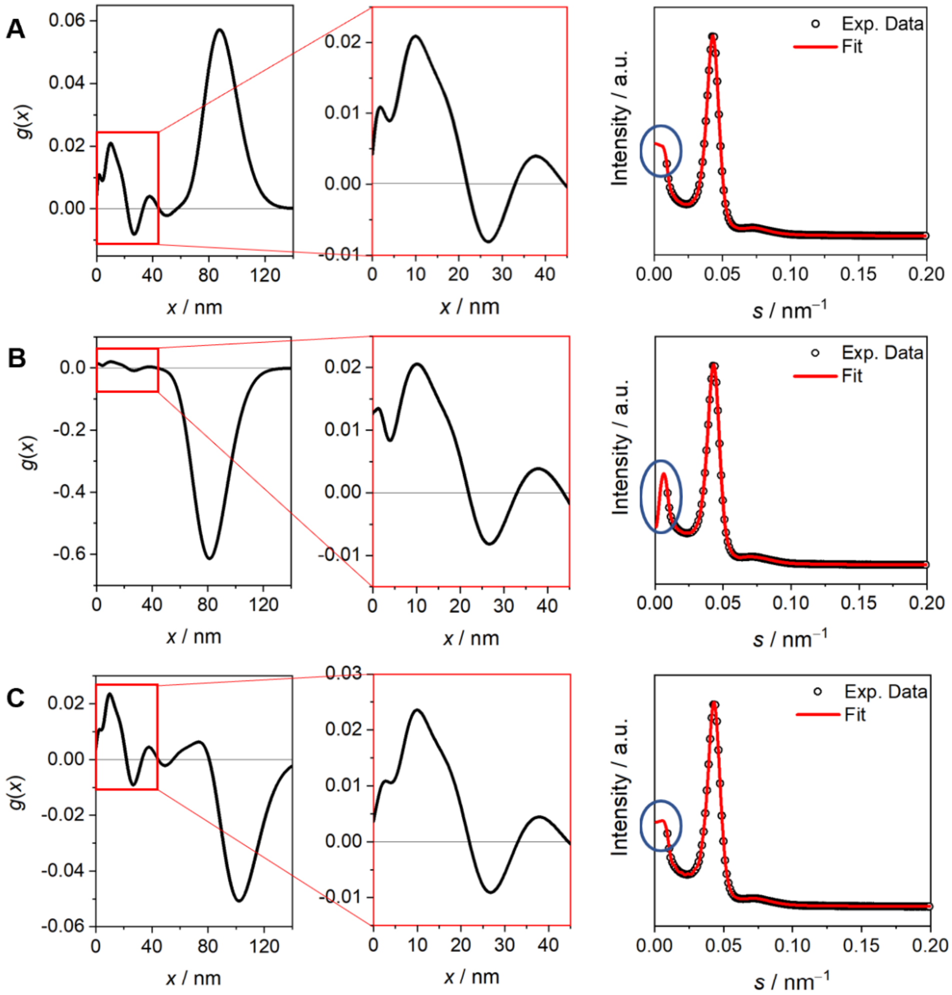

Figure S13. CLD artefact discussion occurring in the SAXS data evaluation at the example of PEO_{259}-*b*-PHA_{95}-templated carbon. Varying two fitting parameters for small scattering vectors s as (A) Tikhonov regularization parameter for small $s = 8.00 \cdot 10^{-4}$ and b (correction parameter due to the data error) = 2.10, (B) Tikhonov regularization parameter for small $s = 8.00 \cdot 10^{-5}$ and b = 2.10, and (C) Tikhonov regularization parameter for small $s = 8.00 \cdot 10^{-4}$ and b = 2.35 effects position and intensity of the artefact in the CLD (left) and fitting withing the scattering pattern (right). For details of this procedure please refer to Smarsly *et al.*[8] and a software tool available from the authors.

The pronounced artefact in the CLD at larger *r* is due to missing data points in the scattering pattern at very small *s* causing varying fitting curves only depending on chosen fitting parameters at small *s* (Tikhonov regularization parameter for small *s* and a correction parameter due to the data error). Due to the artifact, intensities do not strive towards zero in the shown section of the CLD (**Figure S13** left). However, the artifact does not superimpose the distribution´s crucial part for structure evaluation of present mesoporous carbons. Without mathematical specification in the form of a data point, the fitting function at small *s* is undefined by experimental data. Small changes in above mentioned parameter values do not influence the overall fitting and CLD in the crucial mesopore range in question (as shown in the middle column in **Figure S13**) but only effects position, direction, and intensity of the artefact in the CLD (**Figure S13** left) and fitting curve at small *s* (**Figure S13** right).

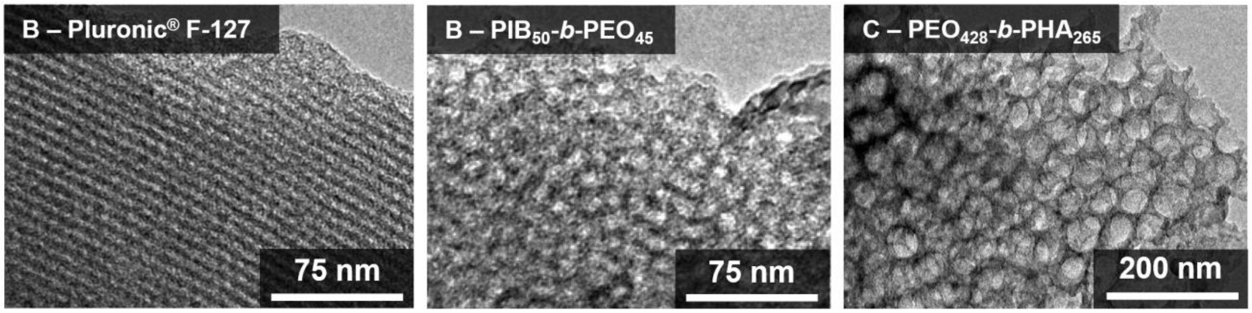

Figure S14. TEM images of (A) Pluronic® F-127-templated carbon, (B) PIB_{50}-*b*-PEO_{45}-templated carbon, and (C) PEO_{428}-*b*-PHA_{265}-templated carbon carbonized at 1300 °C exhibiting the same pore size as carbonized at 900 °C (**Figure 2**).

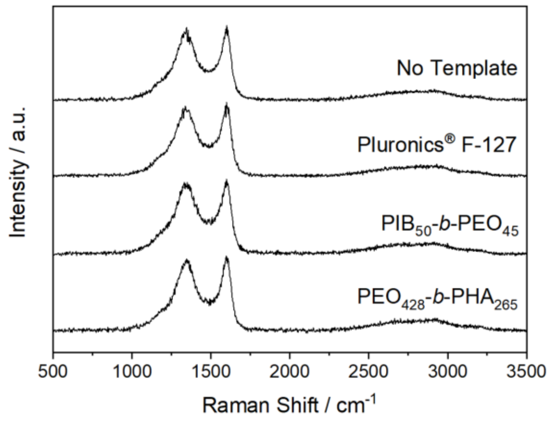

Figure S15. Raman overview spectra of (from top to bottom) non-templated carbon, Pluronic® F-127-templated carbon, PIB_{50}-*b*-PEO_{45}-templated carbon, PEO_{428}-*b*-PHA_{265}-templated carbon carbonized at 900 °C.

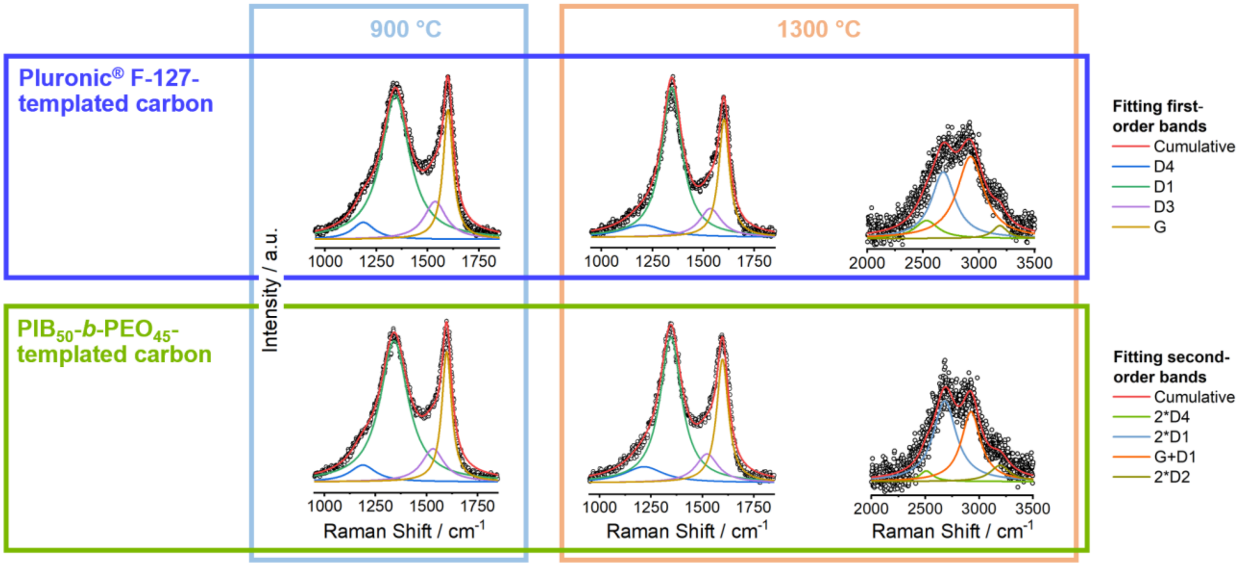

Figure S16. Additional fittings of first- and second-order Raman bands of Pluronic® F-127-templated carbon (dark blue) resp. PIB_{50}-*b*-PEO_{45}-templated carbon (green) carbonized at 900 °C (light blue) resp. 1300 °C (orange).

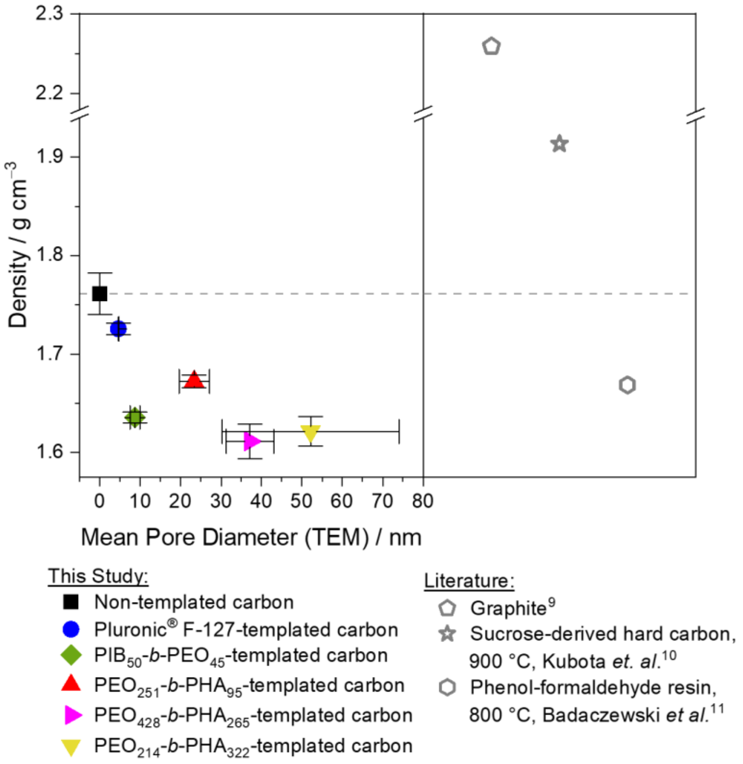

Figure S17. Densities determined by helium pycnometry measurements of non-templated cabon, Pluronic® F-127-templated carbon, PIB$_{50}$-*b*-PEO$_{45}$-templated carbon, and PEO$_n$-*b*-PHA$_m$-templated carbons, carbonized at 900 °C in comparison to literature density values of graphite[9], sucrose-derived hard carbon by Kubota *et al.*[10] and phenol-formaldehyde resin by Badaczewski *et al.*[11]

A low density is an intrinsic property of glassy carbons which is well known in literature due to inaccessible porosity.[11,12] That the determined low density is not only caused by a larger interlayer spacing withing turbostratic carbon compared to graphite can be shown by a rough calculation. Graphite exhibits a density of 2.26 g cm^{-3} with an interlayer spacing of 3.35 Å.[9,13] In this study, for our non-templated carbon carbonized at 900 °C we measured a density of 1.76 ± 0.02 g cm^{-3} and an interlayer spacing a_3 = 3.56 Å. As the percentage increase in interlayer distance does not correspond to the percentage decrease in the density, small voids being

inaccessible for helium contribute to the low density value even for non-templated carbon. As the microstructure analysis revealed, templated carbons carbonized at 900 °C do all exhibit comparable microstructure parameters. Hence, regarding the microstructure, similar density values as for the non-templated carbon samples are expected (dashed line **Figure S16**). Though, densities continue to decrease as large mesopores are introduced. Therefore, isolated mesopores mostly present in PEO_n-*b*-PHA_m-templated carbons are additional contributions lowering the density determined by helium pycnometry, next to the increased interlayer spacing being an intrinsic property of glassy carbons. A decreasing density by introduction of polymer templates also confirms the proposed synthetic mechanism. Crosslinking at moderate temperatures builds a loose network and enables template removal without collapse of the pore structure. The carbon matrix is compacted afterwards at elevated temperatures closing the access to the pore cavities.

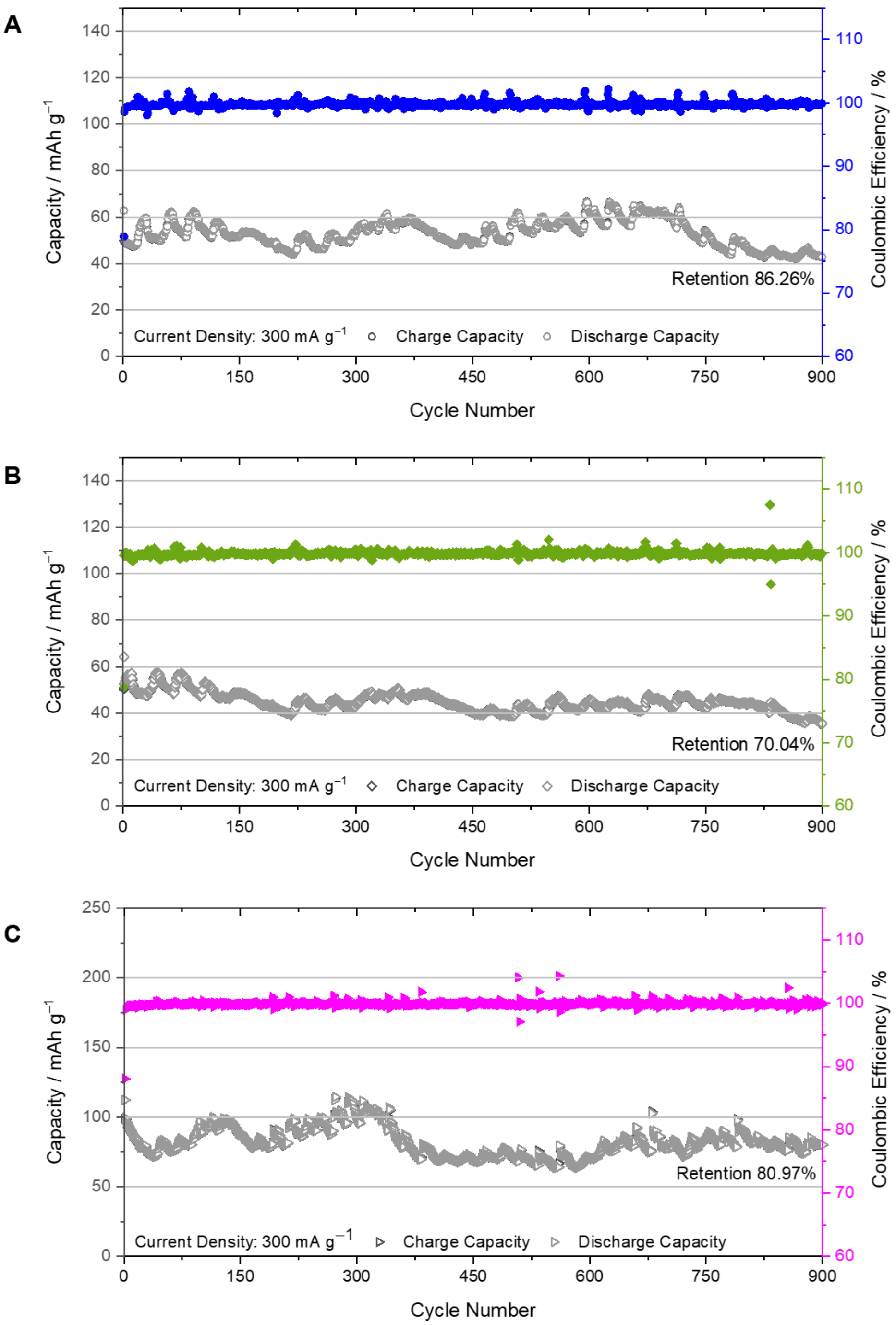

Figure S18. Long-term cycling performance over 900 cycles of sodium-ion batteries with (A) Pluronic® F-127-templated carbon, (B) PIB_{50}-*b*-PEO_{45}-templated carbon, and (C) PEO_{428}-*b*-PHA_{265}-templated carbon as anode materials at 300 mA g^{-1} in a half cell.

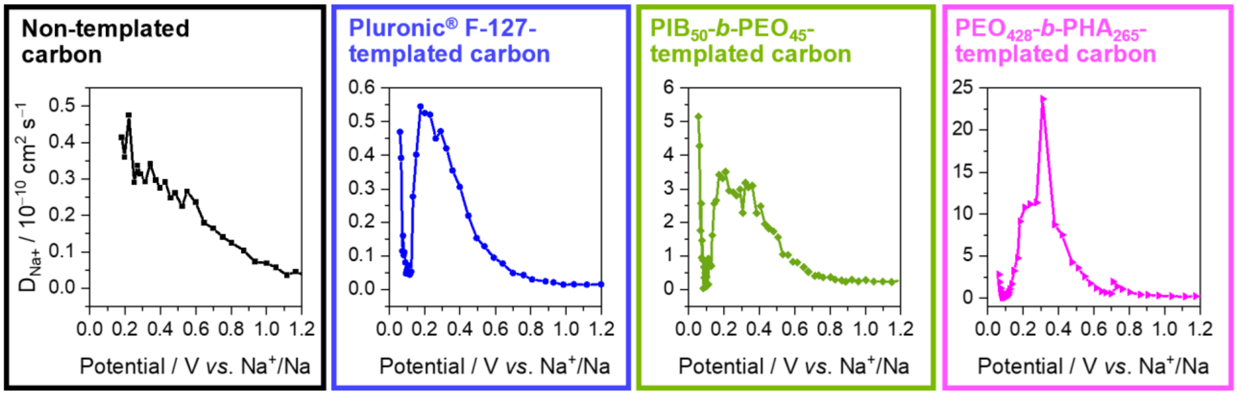

Figure S19. Na^{+} ion diffusion coefficients obtained from GITT tests for desodiation during the second cycle for sodium-ion batteries with non-templated carbon, Pluronic® F-127-templated carbon, PIB_{50}-*b*-PEO_{45}-templated carbon, and PEO_{428}-*b*-PHA_{265}-templated carbon as anode materials.

REFERENCES

(1) Wagner, L. Q.; Prates da Costa; E.; Glatthaar, C.; Breckwoldt, F.; Zecca, M.; Centomo, P.; Huang, X.; Kübel, C.; Schlaad, H.*; et al.* Poly(ethylene oxide)-block-poly(hexyl acrylate) Copolymers as Templates for Large Mesopore Sizes – a Detailed Porosity Analysis. *accepted Chem.Mater.* **2023,** doi.org/10.1021/acs.chemmater.3c01255.

(2) Stoeckel, D.; Wallacher, D.; Zickler, G. A.; Perlich, J.; Tallarek, U.; Smarsly, B. M. Coherent analysis of disordered mesoporous adsorbents using small angle X-ray scattering and physisorption experiments. *Phys. Chem. Chem. Phys.* **2014**, *16*, 6583–6592.

(3) Thomas, A.; Schlaad, H.; Smarsly, B.; Antonietti, M. Replication of Lyotropic Block Copolymer Mesophases into Porous Silica by Nanocasting: Learning about Finer Details of Polymer Self-Assembly. *Langmuir* **2003**, *19*, 4455–4459.

(4) Smarsly, B.; Groenewolt, M.; Antonietti, M. SAXS analysis of mesoporous model materials: a validation of data evaluation techniques to characterize pore size, shape, surface area, and curvature of the interface. *Scattering Methods and the Properties of Polymer Materials*; Springer, Berlin, Heidelberg, 2005; pp 105–113.

(5) Wertheim, M. S. Exact Solution of the Percus-Yevick Integral Equation for Hard Spheres. *Phys. Rev. Lett.* **1963**, *10*, 321–323.

(6) Percus, J. K.; Yevick, G. J. Analysis of Classical Statistical Mechanics by Means of Collective Coordinates. *Phys. Rev.* **1958**, *110*, 1–13.

(7) Smarsly, B.; Göltner, C.; Antonietti, M.; Ruland, W.; Hoinkis, E. SANS Investigation of Nitrogen Sorption in Porous Silica. *J. Phys. Chem. B* **2001**, *105*, 831–840.

(8) Smarsly, B.; Antonietti, M.; Wolff, T. Evaluation of the small-angle x-ray scattering of carbons using parametrization methods. *J. Chem. Phys.* **2002**, *116*, 2618–2627.

(9) Li, Y.; Lu, Y.; Meng, Q.; Jensen, A. C. S.; Zhang, Q.; Zhang, Q.; Tong, Y.; Qi, Y.; Gu, L.; Titirici, M.-M.*; et al.* Regulating Pore Structure of Hierarchical Porous Waste Cork-Derived Hard Carbon Anode for Enhanced Na Storage Performance. *Adv. Energy Mater.* **2019**, *9*, 1902852.

(10) Kubota, K.; Shimadzu, S.; Yabuuchi, N.; Tominaka, S.; Shiraishi, S.; Abreu-Sepulveda, M.; Manivannan, A.; Gotoh, K.; Fukunishi, M.; Dahbi, M.*; et al.* Structural Analysis of Sucrose-Derived Hard Carbon and Correlation with the Electrochemical Properties for Lithium, Sodium, and Potassium Insertion. *Chem. Mater.* **2020**, *32*, 2961–2977.

(11) Badaczewski, F.; Loeh, M. O.; Pfaff, T.; Dobrotka, S.; Wallacher, D.; Clemens, D.; Metz, J.; Smarsly, B. M. Peering into the structural evolution of glass-like carbons derived from

phenolic resin by combining small-angle neutron scattering with an advanced evaluation method for wide-angle X-ray scattering. *Carbon* **2019**, *141*, 169–181.

(12) Badaczewski, F. M.; Loeh, M. O.; Pfaff, T.; Wallacher, D.; Clemens, D.; Smarsly, B. M. An advanced structural characterization of templated meso-macroporous carbon monoliths by small- and wide-angle scattering techniques. *Beilstein J. Nanotechnol.* **2020**, *11*, 310–322.

(13) Chung, D. D. L. Review Graphite. *J. Mater. Sci.* **2002**, *37*, 1475–1489.
